# SgRNA Expression of CRIPSR-Cas9 System Based on MiRNA Polycistrons as a Versatile Tool to Manipulate Multiple and Tissue-Specific Genome Editing

**DOI:** 10.1038/s41598-017-06216-w

**Published:** 2017-07-19

**Authors:** Chen Xie, Yan-Lian Chen, Dong-Fang Wang, Yi-Lin Wang, Tian-Peng Zhang, Hui Li, Fu Liang, Yong Zhao, Guang-Ya Zhang

**Affiliations:** 1 College of Chemical Engineering, Huaqiao University, Xiamen 361021 Fujian, China; 2 Key Laboratory of Gene Engineering of the Ministry of Education, Cooperative Innovation Center for High Performance Computing, School of Life Sciences, Sun Yat-sen University, Guangzhou 510006 Guangdong, China; 3 Shenzhen Weiguang Biological Products Co., Ltd, Shenzhen 518107 Guangdong, China; 4 Department of Spine Surgery, Shenzhen People’s Hospital, Jinan University School of Medicine, Shenzhen 518020 Guangdong, China; 5 Biochip Laboratory, Yantai Yuhuangding Hospital Affiliated to Qingdao University, Yantai, 264000 Shandong China

**Keywords:** Biological techniques, Biotechnology

## Abstract

CRISPR/Cas9-mediated genome editing is a next-generation strategy for genetic modifications. Typically, sgRNA is constitutively expressed relying on RNA polymerase III promoters. Polymerase II promoters initiate transcription in a flexible manner, but sgRNAs generated by RNA polymerase II promoter lost their nuclease activity. To express sgRNAs in a tissue-specific fashion and endow CRISPR with more versatile function, a novel system was established in a polycistron, where miRNAs (or shRNAs) and sgRNAs alternately emerged and co-expressed under the control of a single polymerase II promoter. Effective expression and further processing of functional miRNAs and sgRNAs were achieved. The redundant nucleotides adjacent to sgRNA were degraded, and 5′- cap structure was responsible for the compromised nuclease capacity of sgRNA: Cas9 complex. Furthermore, this strategy fulfilled conducting multiplex genome editing, as well as executing neural- specific genome editing and enhancing the proportion of homologous recombination via inhibiting NHEJ pathway by shRNA. In summary, we designed a new construction for efficient expression of sgRNAs with miRNAs (shRNAs) by virtue of RNA polymerase II promoters, which will spur the development of safer, more controllable/regulable and powerful CRISPR/Cas9 system-mediated genome editing in a wide variety of further biomedical applications.

## Introduction

Clustered regularly-interspaced short palindromic repeats associated protein 9 (CRISPR/Cas9), an RNA-guided endonuclease derived from the type II CRISPR-Cas bacterial adaptive immune system, holds a tremendous promise for biomedical research and has been broadly harnessed in genome editing. The modified CRISPR/Cas system is comprised of two components, the CRISPR associated protein endonuclease 9 (Cas9), and the chimeric crRNA-tracrRNA, termed the single guide RNA (sgRNA)^1^. Typically, Cas9 is synthesized in eukaryotic cells by RNA polymerase II promoter, which can be readily modulated in a tissue- specific or inducible manner, while expression of sgRNA most commonly relies on RNA polymerase III promoter, especially, the U6 promoter. However, this type of promoters do not possess the ability to expression in a tissue-specific way, which is a drawback when tissue-specific genome editing were performed.

The expression of multiple sgRNAs is indispensable in multiplex genome editing^1^, particularly, in the clinical application of Cas9 nickase genome editing in gene therapy for its high specificity that can reduce uncertain risk from host-genome integration^2^. Another example is that CRISPR/Cas system has been also developed as a useful tool for RNA-guided regulation of transcription in eukaryotes, suggesting it is feasible to simultaneously regulate different gene expressions by separately using diverse modulatory methods including up-regulation, down-regulation, and knockout^2–4^. Although these strategies sound interesting and promising, a tedious procedure of design and operation, especially the need to express multiple sgRNAs limits their extensive application. Currently, the synthesis of a series of sgRNAs mainly depends on multiple U6 promoters located in one or more plasmids^5^, in which, each promoter is in charge of only one RNA-based effector. However, this may cause several problems. For instance, repetition of H1 promoters in a lentiviral vector leads to a high rate of recombination and/or deletion of the RNA expression cassettes^6^. Consequently, a simpler and versatile construction is urged to express multiple sgRNAs and additionally, such construction must be sensitive to multiplex regulation.

For a rigorous *in vivo* genome editing, it is necessary to ensure the perfect specificity **-** minimal off-target events and maximal tissue specificity **-** especially in clinical research. An array of strategies were successively developed to avoid off-target effect mediated by CRISPR/Cas system such as double nickase Cas9 nuclease^3^, Cas9 fusion with ZFN^7^, and high-fidelity variant Cas9^8^. On the other hand, tissue-specific promoters and carriers have also been introduced, allowing the specific expression of Cas9, to some extent, in a particular population of cells. However, all these technologies are still insufficient to maintain high tissue specificity, since we can barely find a tissue- specific promoter that is only active in a certain type of cells. For example, CaMKII promoter is widely used to drive the expression of indicated gene in a neural-specific manner^9^. However, it is also active in cardiomyocytes^10^, epithelia^11^, T-cells^12^ *etc*. Low tissue specificity generates potential problem for *in vivo* application of such promoters. Consequently, expressing Cas9 protein together with multiple sgRNAs using different promoters that can be activated only in a given cell type is an attractive strategy, which can ensure that CRISPR system is operative only in a population of highly specific cells, rather than an entire tissue or organ.

As described above, sgRNA is commonly produced under the control of RNA polymerase III promoters^1, 13, 14^. These promoters are effective and ubiquitously active to produce RNA transcripts with definite start and stop sequences without special structure such as intron, 5′ cap, or 3′ poly (A) tail, thereby suited for short RNA transcription^15^. However, functions of RNAs generated by RNA polymerase III are always limited by their short half-life and limited length. While sgRNAs driven by polymerase II promoters has a variety of advantages, which enable coordinated and inducible modulation over multiple aspects of cellular behavior, as well as production of multiple sgRNAs from a single transcript^16^. For these reasons, here we describe a new strategy to express multiple sgRNAs in company with several miRNAs (shRNAs) from a single transcript under the control of an RNA polymerase II promoter. In this method, miRNAs (or shRNAs) and sgRNAs alternately emerged in a polycistron driven by a single RNA polymerase II promoter. The polycistron was processed by the microprocessor - a protein complex containing an RNase III enzyme, Drosha, and its cofactor, DGCR8/Pasha - to form mature sgRNAs and miRNAs (or shRNAs). Furthermore, we found that 5′ cap structure could destroy the nuclease activity of sgRNA: Cas9 complex. At last, this novel strategy is expected to be employed in fulfilling tissue-specific genome editing, multiplex genome editing, genome editing mediated by Cas9 nickase and RNA-guided regulation of gene expression in eukaryotes.

## Results

### RNA Polymerase II promoter cannot be directly used to express sgRNA

**Figure 1:**
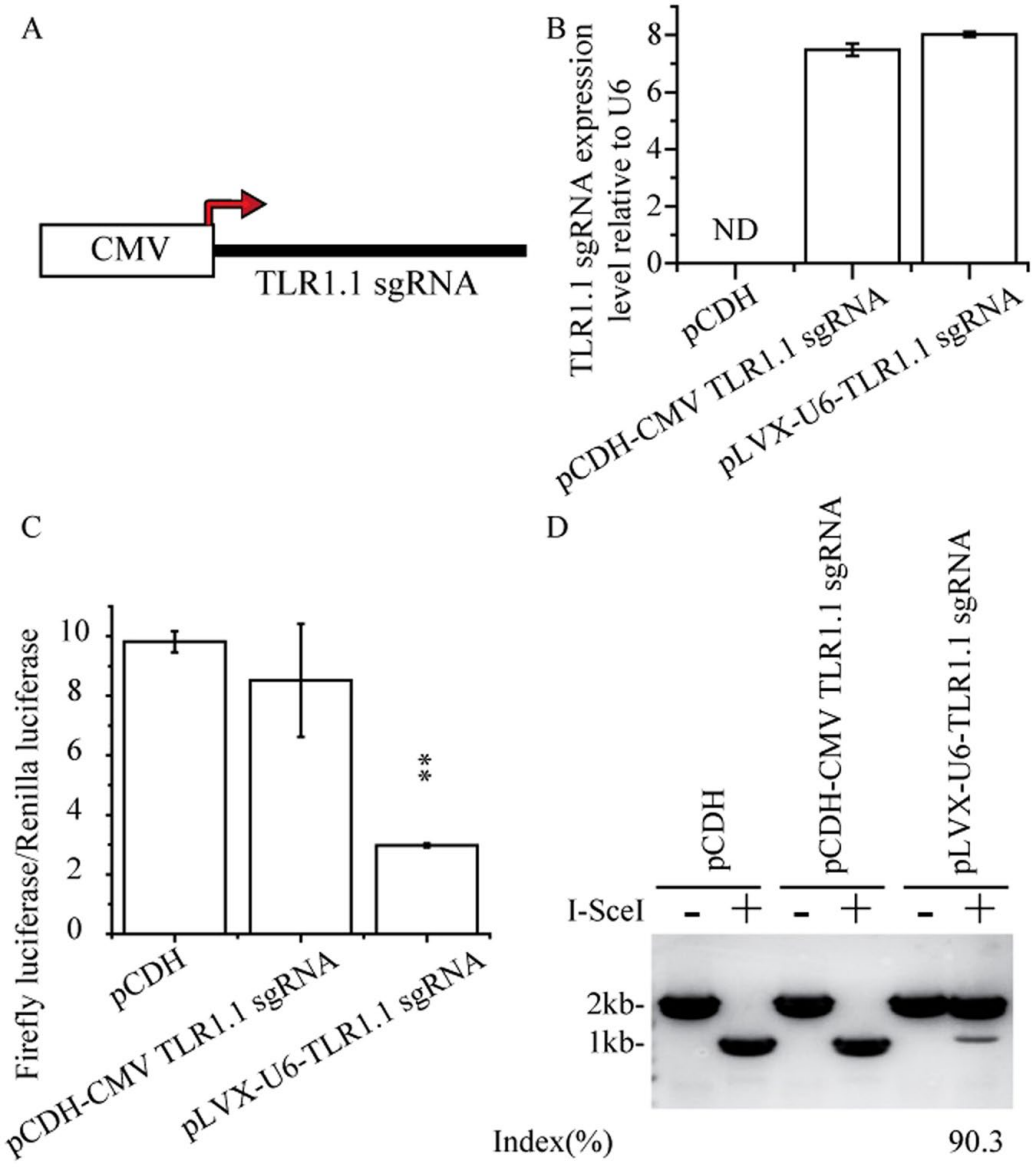
RNA polymerase II promoter cannot be directly used to express sgRNA. (**A**) Designment and the structure of CMV-TLR1.1 sgRNA cassettes. TLR1.1 sgRNA was inserted into pCDH-CMV-MCS-EF1-copGFP vector at indicated positions to construct pCDH-CMV-TLR1.1 sgRNA cassettes. (**B**) qRT-PCR showed that the expression level of TLR1.1 sgRNA transcribed by CMV promoter was comparable to that transcribed by U6 promoter. ND represents no detectable PCR products. (**C**,**D**) sgRNAs directly transcribed by CMV promoter lacked the nuclease activity. The nuclease activity of sgRNA transcribed using U6 and CMV promoter was respectively evaluated by luciferase assay (**C**) and restriction endonuclease digestion assay (**D**).

As described previously, sgRNA is synthesized by RNA polymerase III promoters *in vivo*. We wonder if RNA polymerase II promoter could also be used to generate sgRNA. First, we designed a sgRNA targeting to an I-SceI nuclease sites of TLR1.1 reporter plasmid^17^, termed as TLR1.1 sgRNA, and inserted it at downstream of CMV promoter which is one kind of ubiquitously active polymerase II promoter (Fig. 1A). We also constructed a luciferase based reporter named pGL3-control NHEJ V2 to evaluate the efficiency of nuclease-induced genome engineering in human cell lines. The pGL3-control NHEJ V2 contains a TLR1.1 sgRNA target site and a T2A peptides at the upstream of firefly luciferase. In this system, I-SceI (sgRNA) generates double strand breaks at embedded nuclease cleavage site, leads to activation of NHEJ-mediated DNA damage response (DDR). As a result, firefly luciferase will be inactived due to open reading frame (ORF) shift or fused firefly luciferase product caused by breakdown of T2A peptides. The reduced firefly luciferase activity was measured to indicate the nuclease activity produced by complex components of a variety of TLR1.1 sgRNA and Cas9. We found that the expression level of TLR1.1 sgRNA produced by CMV promoter was comparable to that using U6 promoter (Fig. 1B). Furthermore, we co-transfected sgRNA-Cas9 expression construction and pGL3-control NHEJ V2 reporter into 293T cells. Dual-luciferase (Fig. 1C) and I-SceI nuclease digestion assay (Fig. 1D) were performed to determine the efficiency of genome editing mediated by CMV TLR1.1 sgRNA. Unfortunately, CMV TLR1.1 sgRNA possessed no nuclease activity, suggesting that CMV promoter was not suitable to express sgRNA directly. These results were also confirmed by TLR1.1 reporter system and T7E1 assay (data no shown).

### Design and construction of miRNA- based sgRNA cassettes

**Figure 2:**
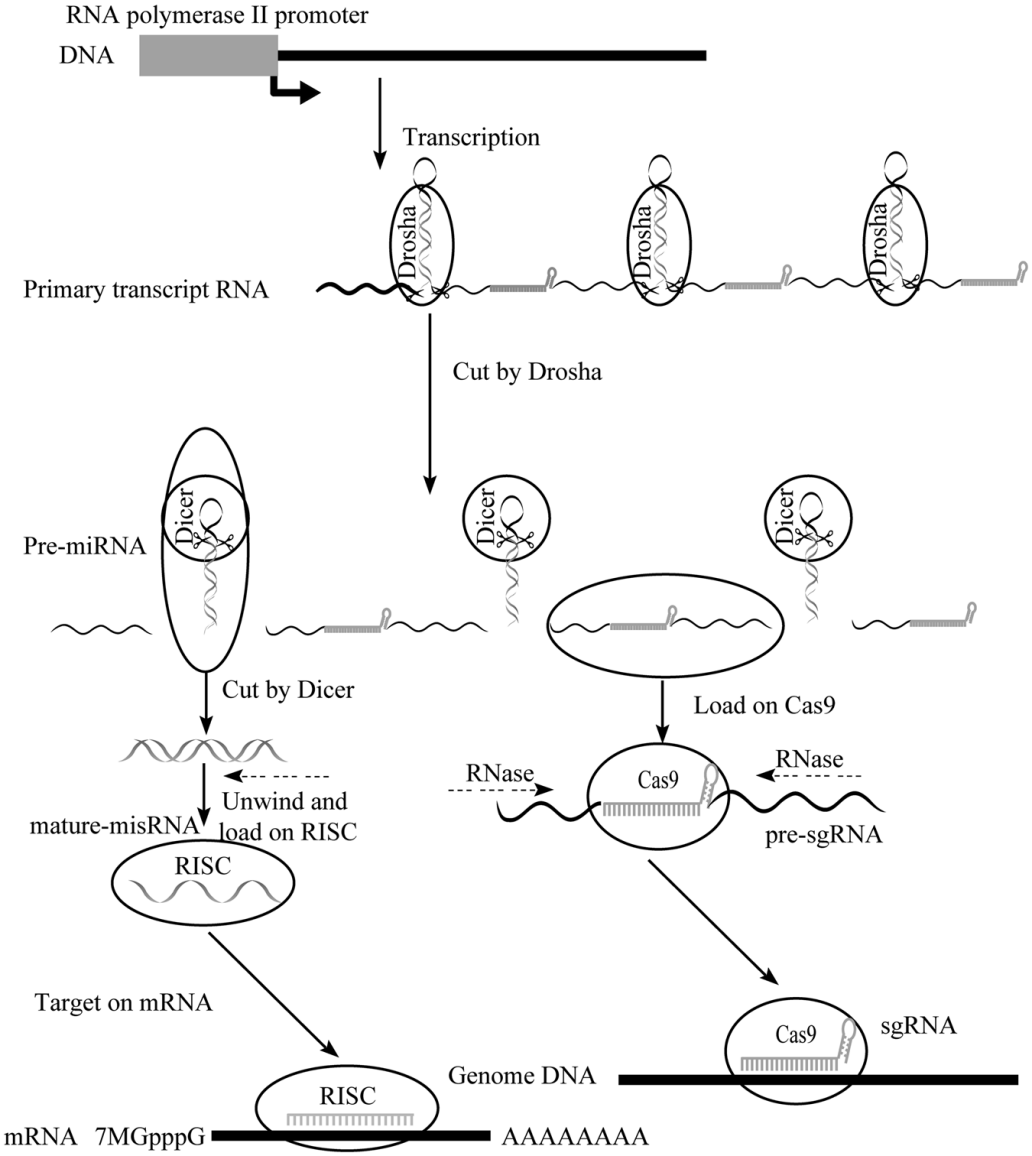
Schematic overview of the miRNA-based CRISPR vector construction system for multiplex genome engineering. In this construction miRNAs (or shRNAs) and sgRNAs alternately emerge in a polycistron. 5′ and 3′ arm of miRNAs (or shRNAs) were required for Drosha to recognize and cut the pri-miRNAs (or pri-shRNAs) from the original transcription. Pre-miRNA (or pri-shRNAs) can be transported into cytoplasm then cut by Dicer to form a double stranded RNA. Further, these double stranded-RNAs can be loaded onto RISC complex and function as a mature miRNA (or siRNA) to target on its corresponding mRNA. Simultaneously, sgRNAs bind to Cas9 (including dCas9-VP64, dCas9-KRAB, etc.). Redundant nucleotides can be degraded by ubiquitous RNase.

To express sgRNA with RNA polymerase II promoter, we designed a new construct named miRNA-based sgRNA, in which miRNAs (or shRNAs) and sgRNAs alternately emerged in a polycistron driven by RNA polymerase II or III (Fig. 2). The stem-loop primary transcripts are processed into mature miRNAs (or shRNAs) and sgRNAs by Drosha and Dicer. During the first processing step occurring in the nucleus, the stem-loop from the remainder of the transcript is excised by Drosha to create a series of ~70-nucleotide pre-miRNA products. Meanwhile, sgRNA is released and forms functional complex with Cas9. Redundant nucleotides in pre-sgRNA can be degraded by ubiquitous RNase^2, 3^. The aforementioned pre-miRNA is transported from the nucleus to the cytoplasm by RAN–GTP and Exportin 5, and subsequently cleaved by Dicer to generate a ~20-nt miRNA: miRNA* duplex. Afterwards, one strand of this duplex is assembled into the RNA-induced silencing complex (RISC), while another strand is degraded. The mature miRNA (or shRNA) binds to its mRNA target site through base pairing and then negatively regulate gene expression^18^ (Fig. 2).

Processing depends on the sequence of pri-miRNA, which folds into a stem loop structure with a terminal loop and flanking segments. However, effective miRNA processing cannot be ensured merely by the formation of stem loop structure. A typical animal pri-miRNA usually consists of an imperfectly paired stem of about 33 nt, a miRNA duplex of ~22 nt, and an 11 nt basal stem flanked by pre-miRNA hairpins, which is necessary for further processing^19^. Besides, additional criteria are also important for effective miRNA (or shRNA) processing, such as an UG motif located at 14 nt upstream of 5p cleavage site of Drosha, an SRp20-binding motif (CNNC motif) resided at about 18nt downstream of 3p cleavage site of Drosha, an UGUG motif in the apical stem, and at least 9 unstructured nucleotides flanking both sides of basal stem, etc.^19^. In order to fulfill efficient ectopic expression of miRNAs (shRNAs) and sgRNAs, additional sequences within ~40 nt upstream and ~40 nt downstream of the pre-miRNA hairpins in natural or artificial animal miRNA arm were required for recognition and cleavage by Drosha and Dicer from the original transcript^19^.

Of note, these constructions were remarkably characterized by the following advantages: (1) multiple sgRNAs can be generated by using one single promoter for genome editing; (2) miRNAs (or shRNAs) and sgRNAs can be produced to knock down and knock out specified genes simultaneously; (3) both RNA polymerase II and III promoters can be used to express these miRNA-based sgRNA; (4) tissue-specific promoters can be employed to drive the expression of sgRNA, which is powerful to enhance the cell type- and/or tissue-specificity of genome editing; (5) these constructions possess shorter sequences compared to those generated by conventional methods^2–4^, and can avoid the presence of multiplex promoters that is harmful in multi-target genome editing; (6) the short sequences make it feasible to achieve multiple genome editing *in vivo* by using a single Adeno-associated virus (AAV) vector, which is a superb vehicle for its low immunogenic potential, reduced oncogenic risk due to host-genome integration^20^, and broad range of serotype specificity^21^. Nevertheless, the restrictive cargo size (4.5 kb excluding the inverted terminal repeats) of AAV presents a seemingly intractable obstacle for packaging the commonly used Cas9 (3~4 kb), multiple guide RNAs (sgRNA, ~120 bp each), and corresponding promoters (hundreds of bp each) together in a single vector.

### miRNA-based sgRNA cassettes express functional sgRNAs and miRNAs simultaneously. 

Before applying miRNA-based sgRNA to genome editing and RNAi simultaneously, some issues need to be elucidated. Firstly, whether the miRNA-based sgRNA strategy could create functional sgRNA and miRNAs (or shRNAs). Secondly, RNAs transcribed by polymerase II promoter are characterized by cap at 5′ end, polyadenylations at 3′ end and unknown structures, which might interfere with activity and function of sgRNA:Cas9 complex. Thirdly, whether sgRNAs interfere the expression of miRNAs.

**Figure 3:**
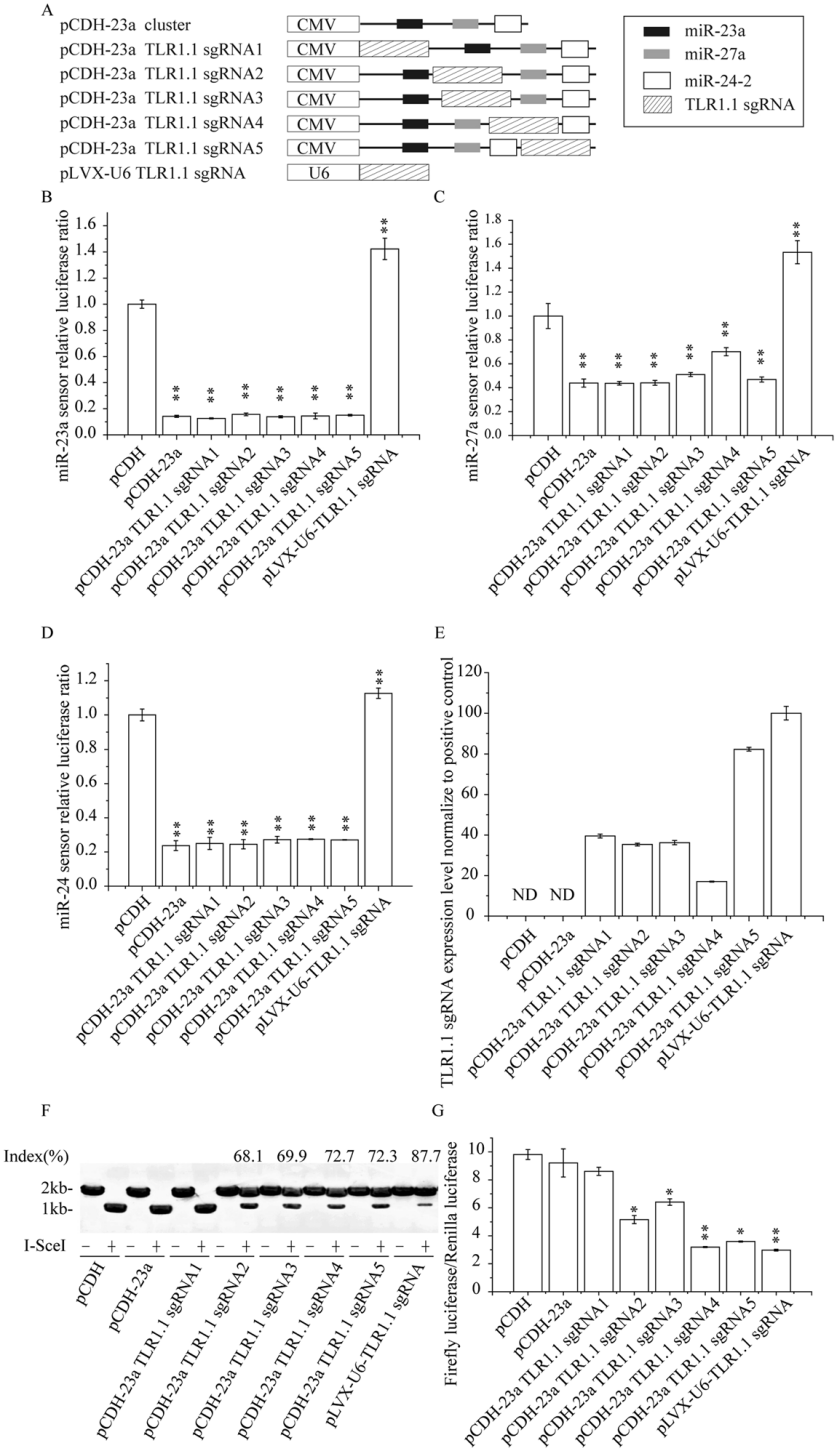
Polycistronic miRNA-based sgRNA construction produced active miRNA and sgRNA. (**A**) Designment and structure of miR-23a/27a/24-2 cluster based TLR1.1 sgRNA cassettes. TLR1.1 sgRNA was inserted into miR-23a/27a/24-2 cluster at indicated positions to construct five different miR-23a/27a/24-2 cluster based TLR1.1 sgRNA constructions. Based on the insertion position, these five sgRNAs constructions were respectively termed as pCDH-23a TLR1.1 sgRNA1, 2, 3, 4, 5. (**B**–**D**) miRNA sensors were used to detect the expression of miR-23a (**B**), miR-27a (**C**) and miR-24-2 (**D**). 293T cells were transfected with miRNA luciferase sensor constructions containing the sequence targeted by indicated miRNA as well as miRNA-based sgRNA constructions described above. Cells co-transfected with the reporter plasmid and empty vector (pCDH) served as a negative control, while cells co-transfected with the reporter plasmid and miR-23a cluster expression vector (pCDH-23a cluster) served as a positive control. Silencing activity was measured as the ratio of Renilla to firefly luciferase activity in cell lysates at 48 h after transfection, and results were normalized to corresponding negative control. Data were represented as the mean ± standard deviation (SD) of triplicate assays. (**E**) qRT-PCR were performed to evaluate the expression level of mature sgRNA. ND represents no detectable PCR products after 30 cycles. (**F**,**G**) miRNA based sgRNA expressed with CMV promoter can be used as a tool for genome editing. Indicated miRNA based sgRNA expression vector, Cas9 expression vector, pGL3-control NHEJ V2 vector (containing an I-SceI nuclease/TLR1.1 sgRNA target site) and pRL-TK vector were co-transfected into 293 T cell. Six days after transfection, the cells were harvested and then used as temples for I-SceI digestion assay (**F**) and luciferase reporter assay (**G**) to evaluate the nuclease activity of sgRNAs driven by different plasmids.

To elucidate these issues, we constructed a series of miRNA-based TLR1.1 sgRNAs by inserting a TLR1.1 sgRNA at different positions in human miR-23a/miR-27a/miR-24-2 cluster gene (Fig. 3A). qRT-PCR result demonstrated that all these miRNA-based sgRNAs vectors expressed the miRNA cluster and TLR1.1 sgRNA efficiently (Figure S1 and Fig. 3E). Further, we expressed Cas9 and pGL3-control NHEJ V2 reporter with or without the above miRNA-based TLR1.1 sgRNAs in 293T cells and analyzed with I-SceI nuclease digestion assay (Fig. 3F), dual-luciferase assay (Fig. 3G) and T7E1 assay (date not shown). All of the results show that miRNA-based TLR1.1 sgRNAs, except TLR1.1 sgRNA1, can express sgRNA with nuclease activity.

To better assess the effect of sgRNA insertions on miRNA activity, miRNA sensors were used to measure the efficiency of different vectors in expressing miRNAs. miRNA sensor serves as a tool to profile the activity of miRNA by inserting the corresponding miRNA targets into the 3′ UTR of Renilla luciferase^22, 23^. This system detects not only the level of miRNA expression but also the activity of the miRNA. Besides, firefly luciferase is used as an internal control to get rid of artifact effects caused by cell line viabilities, and experimental bias. In this experiment, miRNA-based TLR1.1 sgRNAs were separately co-transfected with a library of miRNA sensor vectors, such as miR-23a, miR-27a and miR-24, respectively. The results showed that most of TLR1.1 sgRNA insertion did not significantly influence the expression of miR-23a, miR-27a and miR-24. But TLR1.1 sgRNA4 which inserted between miR-27a and miR-24-2 could slightly inhibit the expression of miR-27a (Fig. 3B–D).

For TLR1.1 sgRNA1 construction, TLR1.1 sgRNA was inserted at the upstream of miR-23a, implying that sgRNAs processed from this construction harbored a cap structure. Taking into account the fact that RNA polymerase II promoter cannot produce functional sgRNAs directly, we hypothesized that the cap structure might destroy the nuclease function of sgRNA whereas polyA tail could not adversely affect its activity. Northern blot results showed that sgRNAs located at the upstream of miR-23a exhibited greater molecular weight than other mature sgRNAs (Figure S2), suggesting that Cas9 bound onto sgRNA sequence to avoid the degradation of sgRNA from endogenous RNase, and on the other hand the cap structure protected the redundant sequences at 5′ ends of sgRNA from being degraded by ubiquitous RNase. Collectively, we can draw a conclusion that cap structure did restrain the sgRNA: Cas9 nuclease activity, which may explain why RNA polymerase II could not be used to express functional sgRNAs directly.

### miRNA - based sgRNA cassettes serve as a versatile tool for genome editing

**Figure 4:**
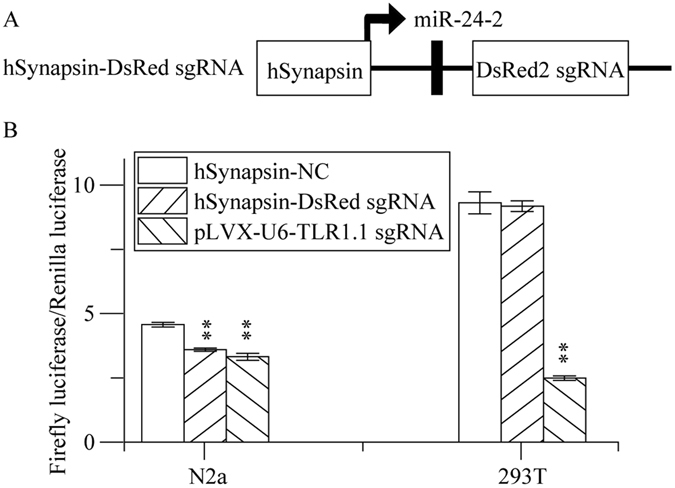
MiRNA based sgRNA cassettes serve as a useful tool for tissue specific genome editing. (**A**) Schematic overview of construction. (**B**) miRNA based sgRNA can be modified to adapt tissue specific genome editing. Briefly, DsRed2 sgRNA was inserted after miR-24-2 and driven by hSynapsin promoter, a neural specify promoter. DsRed2 sgRNA driven by hSynapsin was co-transfected with Cas9 expression vector, pGL3-control NHEJ V2 reporter vector, and pRL-TK into 293T kidney cells or N2a mouse neural cells. 6 days after transfection, luciferase assay was used to determine the nuclease activity of neuron specific sgRNA driven by hSynapsin. The expression of reporter gene was inhibited in N2a cells instead of 293T cells.

In order to apply miRNA-based sgRNA in tissue-specific genome editing, we designed a constructions in which a DsRed2 sgRNA was adjacent to the downstream of miR-24-2 driven by hSynapsin, a neuron- specific RNA polymerase II promoter implicated in the regulation of neurotransmitter release at synapses (Fig. 4A)^24^. We then expressed this DsRed2 sgRNA with Cas9, pGL3-control NHEJ V2 and pRL-TK in N2a mouse neuron cell line or 293T human kidney cell line. As shown in Fig. 4B, the expression level of firefly luciferase could be was inhibited in N2a cells rather than 293T cells, suggesting that the tissue specificity can be accomplished this way.

**Figure 5:**
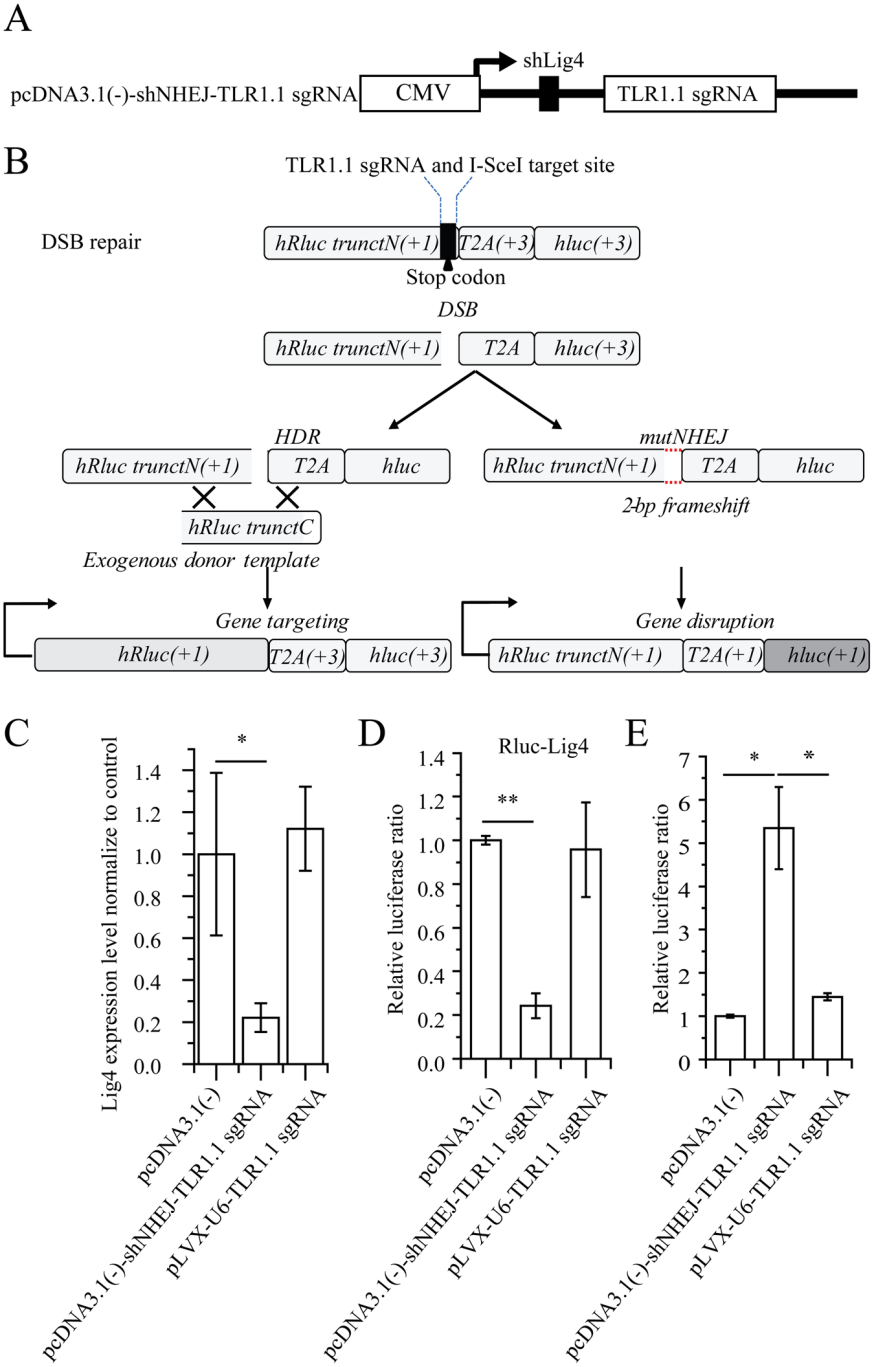
MiRNA based sgRNA cassettes can be modified to alter the proportion of HR in genome editing. (**A**) Schematic overview of pcDNA3.1(−) shNHEJ-TLR1.1 sgRNA construction. (**B**) Schematic overview of the principle of detection of the ratio of HR and NHEJ. qRT-PCR (**C**) and luciferase reporter assay (**D**) were used to demonstrate that Lig4 was effectively suppressed by pcDNA3.1(−) shNHEJ TLR1.1 sgRNA. (**E**) Gene editing with pcDNA3.1 (−) shNHEJ TLR1.1 sgRNA had a higher efficiency of homologous recombination compared to pLVX-u6-TLR1.1 sgRNA. pcDNA3.1(−) shNHEJ TLR1.1 sgRNA or corresponding control was co-transfected into 293T with Cas9 expression plasmid, DSB repair reporter construction, and corresponding donor. Afterwards, luciferase assay was performed 3 days after transfection.

Homologous recombination (HR) and non-homologous end joining (NHEJ) are two major pathways which compete to repair DNA double strand breaks^25^. NHEJ is highly efficient but error-prone, therefore, increasing the proportion of HR in genome editing is a very meaningful work. Previous study show that Lig4 make a great contribution to NHEJ pathway^17^. We wonder if we can ameliorate/improve the efficiency of knock-in by CRISPR/Cas9 through inhibiting NHEJ pathway with miRNA or shRNA to enhance HR frequency. We constructed a plasmid named pcDNA3.1(−) shNHEJ TLR1.1 sgRNA harboring shRNA targeting Lig4 as well as TLR1.1 sgRNAs (Fig. 5A) and a Dual-luciferase based system named *DSB repair* to detect the ratio of HR and NHEJ. Notably, by using this plasmid, we could substantially knock down Lig4, (Fig. 5C,D) and significantly ameliorate the efficiency of HR without detectable NHEJ (Fig. 5E).

**Figure 6:**
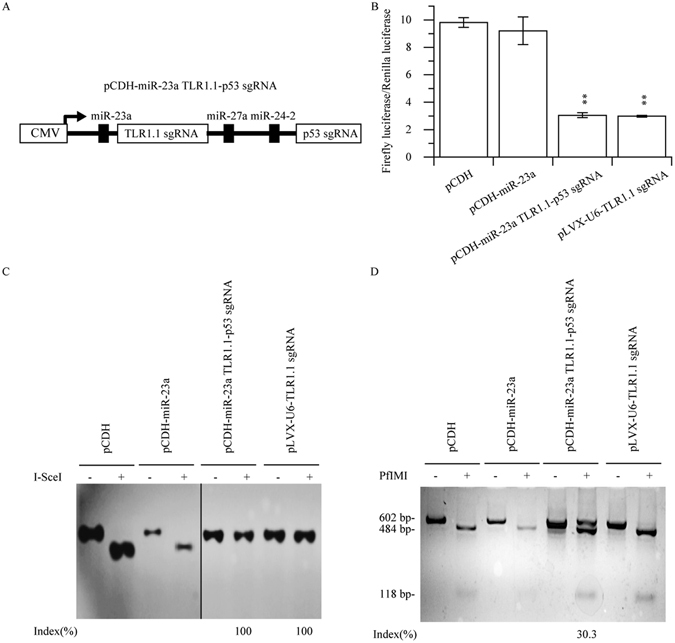
Multiplex genome editing mediated by miRNA based sgRNA. (**A**) Schematic overview of pCDH-miR-23a TLR1.1-p53 sgRNA construction. pCDH-miR-23a TLR1.1-p53 sgRNA or corresponding control was co-transfected into 293T cell with pGL3-control NHEJ V2, pLentiCRISPR, and pRL-TK. 6 days after transfection, cells were harvested in the passive lysis buffer for luciferase assay (**B**). Where a portion of the cell lysate was digested by proteinase K and then served as PCR template in I-SceI nuclease assay (**C**). To demonstrate the nuclease activity of p53 sgRNA, total RNA was extracted from aforementioned cells using Trizol regent, and then was reverse transcribed to serves as a template to amplified p53 cDNA and subjected to PfIMI nuclease assay, which showed that pCDH-miR-23a TLR1.1-p53 sgRNA could serve as a tool to knock out p53.

To confirm whether multiplex sgRNAs can be driven by this strategy, we constructed a plasmid named pcDNA3.1(−) miR-23a TLR1.1-p53 sgRNA harboring two sgRNAs targeting both TLR1.1 reporter and TP53 locus in human genome (Fig. 6A). This plasmid was co-transfected with Cas9, pGL3-control NHEJ V2 and pRL-TK into 293T cells. Notably, both pGL3-control NHEJ V2 (Fig. 6B,C) and human p53 gene were edited (Fig. 6D), demonstrating that this strategy can be used as a tool for multiple genome editing.

## Discussion

Expression of multiplex sgRNA with RNA polymerase II promoter has important application value. A previous study reported a method that was developed to express sgRNA with RNA polymerase II promoter by using self-cleaving ribozymes and tRNA^26^. In addition, sgRNA-shRNA structure has been also introduced to the multiplex genome engineering by using U6 promoter^27^. In this work we have developed a novel miRNA polycistron-based strategy which is driven by an RNA polymerase II promoter, to express multiplex sgRNAs and miRNAs (or shRNAs). Intriguingly, the innovative method exhibited tremendous potential for simultaneously disrupting a variety of gene expressions by employing an individual plasmid and improving tissue specificity of genome editing mediated by CRISPR/Cas9 system. Moreover, this approach allows controllable expression of multiple shRNAs and thus provides a powerful tool to clear virus such as HIV or HBV, since it hampers viral escape from a single shRNA and reduces toxicity from oversaturation of the RNAi machinery^28^.

Our miRNA-based sgRNA cassette strategy for multiple genome editing substantially differs from the traditional one in regarding the transcriptional mechanisms and components: (1) miRNA-based sgRNA can be initiated by both RNA polymerase II and III, whereas conventional sgRNA is only driven by RNA polymerase III; (2) miRNA-based sgRNA is processed by microprocessor so that co-expression of sgRNA and miRNA is feasible; (3) sgRNA is suggested to be inserted between miRNAs or at the end of the cassette; (4) flanking regions of miRNAs are required for the recognition by miRNA processing machinery. As to function, (1) the transcription of multiple sgRNAs and miRNAs can be triggered by a single promoter; (2) integration of diverse sgRNAs and miRNAs in one single vector makes it feasible to edit (or transcriptionally activate) and knock down a myriad of genes efficiently and simultaneously; (3) miRNA-based sgRNA enhances the tissue specificity of genome editing mediated by CRISPR/Cas9. All of these merits discussed above may facilitate its application in complicated and specific genome editing.

In addition, we noticed that both of the sgRNA expressed by pCDH-CMV TLR1.1 sgRNA and pCDH-23a TLR1.1 sgRNA1 were lack of nuclease activity. Since the sgRNAs expressed by the above vectors has a 5′ cap structure, we proved that sgRNA containing a 5′ cap structure could wipe off the activity of sgRNA: Cas9 nuclease. Interestingly, we noticed that sgRNAs with 5′ cap exhibited a molecular weight significantly higher than expected (Figure S2). Taking into account that extended sgRNAs are mostly processed into ~20 nt fragments according to previous documented researches^2, 3^, we proposed that the 5′ cap structure and its binding protein might protect the precursor RNA from degradation thus result in a higher molecular weight. Regarding the mechanism how cap structure abolished the activity of sgRNA: Cas9 complex, we speculated that RNAs containing cap structures were rapidly exported to the cytoplasm, while Cas9 protein mainly retains in nucleus^29^. The efficiency is extremely high when we used sgRNA targeting reporter plasmid (Fig. 6C), however the efficiency of editing endogenous p53 genes is relatively low (Fig. 6D). We hypothesize that this may be due to the interference with nuclease targeting by the complex structure or modification of endogenous genes.

HR is a more precise way than NHEJ in repairing DNA double-strand breaks, however, it only occurs at S/G2 phase, and the low frequency restricts its application. In our study, we developed a novel strategy to enhance the efficiency of HR mediated by CRISPR/Cas9 system, which substantially ameliorate the availability of HR and showed enormous potential.

A scabrous problem with this strategy so far is that construction of the plasmid is somehow complicated and time consuming. Fortunately, the progression of commercial gene synthesis service endows the synthesis of this carrier with time- and cost-efficiencies. Besides, our strategy endows CRISPR with versatile features, and thus advanced biomedical researches will be underpinned by the delicate design. As elucidated, miRNA-based sgRNA could be controlled by tissue-specific promoters, simultaneously suppressing the expression of some tissue-specific genes and fulfilling multiplex genome editing, all of which were expected to tremendously expand the application of CRISPR/Cas9 system. Moreover, this strategy can also play a pivotal role in confronting virus or cancers. For instance, CRISPR sgRNA can be designed to target HPV virus genome integrated in human cervical cancer cells and meanwhile, shRNA or miRNA can target to cells containing unrepaired DSB and induce them apoptosis. Thus, double tissue-specific promoters allow miRNA-sgRNA and Cas9 to specifically and functionally express in cervical cancer cells, which makes this strategy much safer.

In summary, this study provides a new efficient approach of CRISPR/Cas9 for a wide variety of applications: multiplex genome/epigenome editing, simultaneous activation/repression of multiple genes, etc. However, additional investigation is needed to be carried out for further improving the miRNA-based sgRNA system. For instance, engineering modification of vectors for expression of miRNA and sgRNA should be optimized, and we expect to establish double tissue- specific promoter systems to separately express Cas9 and sgRNA and precisely target to a specific cell type, etc. Since most cerebral regions contain different divisions but often share the same biomarker, it was challenging to generate a mouse with a single gene knockout in one particular region of brain, like hippocampus. We believe our novel strategy may feature prominently in achieving this and contribute a lot to current neurobiological research.

## Materials and Methods

### Plasmid construction

To generate a series of plasmids pCDH-CMV-TLR1.1 sgRNA, pCDH-miR-23a cluster, and pCDH-miR-23a TLR1.1 sgRNA1-5, the corresponding gene sequences were inserted into a pCDH-CMV-MCS-EF1-copGFP vector (cat# CD511B-1, SBI, System Biosciences, Mountain View, CA). As such, pLVX-U6-TLR1.1 sgRNA was obtained by cloning the gene fragment into a pLVX-shRNA2 vector (Clontech, Palo Alto, CA, USA).

In order to construct miRNA sensor vectors, sequences corresponding to the miRNA target sites were cloned into the psiCHECK-2 vector (Promega, Madison, WI, USA) at the downstream of Renilla luciferase. More detail about the primers and methods used in plasmid construction were depicted in Supplemental Information (Table S1).

Regarding the generation of pcDNA3.1 (−) 23a TLR1.1 sgRNA, pCDH-miR-23a TLR1.1 sgRNA2 was digested by XbaI and BamHI, and the procreant miR-23a TLR1.1 sgRNA2 gene fragment was then ligated with linearized pcDNA3.1 (−) vector. Meanwhile, DsRed sgRNA was synthesized and then subcloned into AAV-hSynapsin vector, and shNHEJ TLR1.1 sgRNA was produced and inserted into a pcDNA3.1 (−) vector.

pGL3-control NHEJ V2 was modified from pGL3-control (Promega, Madison, WI, USA) to measure the efficiency of genome editing. Specially, TLR1.1 sgRNA target site, a portion of DsRed2 gene and a T2a element were fused with firefly luciferase within the same open reading frames. Both TLR1.1 sgRNA and DsRed2 sgRNA, associated with Cas9, can destroy the activity of firefly luciferase. DSB repair was modified from psicheck2 (Promega, Madison, WI, USA) to evaluate the change in the ratio between HR and NHEJ (Fig. 5A).

All sequences were synthesized by *Oligobio* Co., Ltd (Beijing, China), and detail information was provided in supplemental data.

### Cell culture

HEK 293T cell lines were cultured in DMEM high glucose medium (Invitrogen, Carlsbad, CA, USA) and Neuro-2a were maintained in MEM medium (Invitrogen, Carlsbad, CA, USA) supplemented with 10% (V/V) fetal bovine serum (FBS, Invitrogen, Carlsbad, CA, USA), antibiotics (100 U/mL of penicillin and 100 µg/ml of streptomycin, Invitrogen, Carlsbad, CA, USA). Cultures were incubated in a humidified condition with 5% CO_2_ at 37 °C.

### Transfection

PEI applied to the transfection of 293T cells while lipo2000 was accustomed to both 293T cell and N2a cells. In detail, for transfections involving Northern blotting assay, 80% confluent 293T cells were seeded onto a 10 cm dish at a 1:5 ratio, 24 h prior to transfection. A mixture of 7.5 μg Cas9 plasmid (pLentiCRISPR, Plasmid #49535, Addgene) and 7.5 μg of miRNA- based sgRNA plasmid was transfected by using 1 mg/ml PEI (#23966, Polyscience Inc.,) following the manufacturer’s instructions. 15 μg of plasmid DNA was diluted in 1 ml opti-MEM medium (Invitrogen, Carlsbad, CA, USA), before 45 μl PEI solution was added. The contents were mixed thoroughly and incubated at room temperature for 25 min to create the transfection complexes, which were then delivered into cells.

### RNA Extraction and Real-time Quantitative RT-PCR

Total RNA was extracted from cells using the Trizol reagent (Invitrogen, Carlsbad, CA, USA) according to the manufacturer’s instructions. After RNA was quantified and checked for purity and integrity by spectrophotometry at 260 and 280 nm, the cDNA was synthesized through reverse-transcription reaction with Rever-Tra-Ace-α-Transcriptase (Toyobo, Tokyo, Japan) and subsequently amplified by PCR using the SYBR® Premix Ex Taq II (Tli RNaseH Plus) (Takara, Tokyo, Japan).

For real-time quantification of microRNAs, stem-loop method was employed^30^. Stem-loop primers and specific primer of U6 snRNA (served as an internal reference gene) were mixed together to reverse-transcribe miR-23a, 27a, 24, and U6, respectively. Then miRNA qRT-PCR was conducted by using a forward primer located in mature miRNA and a reverse primer situated in the stem-loop primer.

For the real-time quantification of mature sgRNA, poly (A) tailing method was applied to avoid the interference of primary transcription. After adding a poly (A) tail onto 500 ng of total RNA, double bases anchor primer was introduced to reverse-transcribe RNA containing a poly (A) tailing. A forward primer located in mature sgRNA and a reverse primer resided in the double bases anchor primer to perform the qRT-PCR analysis.

For mRNA or total sgRNA (mature sgRNA and primary transcription), reverse transcription was performed using random primers. Gene quantities were normalized to that of the housekeeping gene GAPDH. Quantitative PCR was performed on a LightCycler 480 Real-Time PCR system (Roche, Rotkreuz, Switzerland). Relative fold changes of miRNAs or mRNA expression in treated cells against control cells were calculated using the comparative Ct (2^−ΔΔCt^) method. Each reaction was performed in triplicate.

All of the primer sequences used in qRT-PCR were listed in Table S2.

### Dual-luciferase reporter assays

293T cells were plated onto 96-well plates at a density of 2.0 × ${10}^{4}$ cells/100 μl and cultured overnight.

To measure the expression of miR-23a/27a/24-2 and evaluate the efficiency of miRNA based Lig4 shRNA, cells in each well were co-transfected with 100 ng miRNA or shRNA sensor plasmid and 300 ng miRNA-based sgRNA expression plasmid by using PEI. At 48 h after transfection, cells were harvested. In these experiments, firefly luciferase activity was used as an internal reference. A decrease in relative Renilla luciferase activity signified an increase in miRNA activity.

For assessing the efficiency of genome editing mediated by miRNA-based sgRNA, 20 ng pGL3-control NHEJ V2 containg a I-SceI nuclease site within its coding region was co-transfected into 293T cells with 80 ng indicated sgRNA expression vector, 80 ng Cas9 expression vector and 20 ng pRL-TK vector. Three days after transfection, cells were passaged at a ratio of 1:4. Six days after transfection, the cell were harvested using passive lysis buffer for luciferase assay. Here Renilla luciferase activity was used as an internal reference. A reduced activity of firefly luciferase indicated an increased nuclease activity mediated by sgRNA.

In order to evaluate the ratio of HR and NHEJ, 20 ng DSB repair, 20 ng donor, and 80 ng Cas9 expression vector were co-transfected with 80 ng indicated sgRNA expression vector into 293T cells. Three days after transfection, cells were collected in passive lysis buffer for luciferase assay. The activity of Renilla luciferase was positively correlated with the frequency of HR, and the activity of firefly luciferase was positively associated with the frequency of NHEJ. Consequently, the ratio of Renilla/Firefly was positively related to the ratio of HR/NHEJ.

All of the dual-luciferase assays were performed by using the dual-luciferase reporter assay kit (Promega, Madison, WI, USA), according to the manufacturer’s instructions. Each experiment was repeated in triplicate.

### Restriction endonuclease digestion assay

To evaluate the efficiency of genome editing mediated by miRNA-based sgRNA, 10 μl cell lysates prepared for dual-luciferase assay were incubated for 3 h at 37 °C with an appropriate amount of proteinase K. The procreant digested products were used as the template in subsequent PCR assay (exTaq, Takara, Tokyo, Japan) to amplify a 2 kb fragment of pGL3-control NHEJ V2. Afterwards, 6 μl PCR products were directly digested for 30 min at 37 °C with 0.5 μl I-SceI nuclease (Fementas, USA), followed by 1% agarose gel electrophoresis. Wild-type PCR product was digested and yielded two 1 kb fragment (in a band), while the mutation PCR product could not be digested. The efficiency of NHEJ is defined as the ratio of the intensity of the 2 kb-band to the sum intensity of both bands (also named as index).

Unfortunately, we failed to amplify p53 gene from genomic DNA, but amplified it from cDNA. Consequently, cells transfected with pCDH-miR-23a TLR1.1-p53 sgRNA and Cas9 expression vector were used for purification total RNA by using TRIzol (Invitrogen, Carlsbad, CA, USA). After RNA purification, total RNA is then reverse transcribed into cDNA as template and subsequently a 600 bp fragment of p53 gene was amplified. 6 μl PCR product was digested with 0.5 μl PfIMI nuclease (Fermentas Inc., Ontario, Canada) for 30 min at 37 °C prior to 2.5% agarose gel electrophoresis. Wild-type PCR product was digested and yielded a 484 bp and a 118 bp size fragments, while the mutation PCR product could not be digested. The efficiency of genome editing is defined as the ratio of the intensity of the 602 bp-band to the sum intensity of a total of three bands (also named as index).

### Northern blots

15 μg total RNA was mixed with an equal volume of 2× RNA loading buffer, heated to 65 °C for 5 min, chilled on ice for 1 min and then loaded onto 8% denaturing polyacrylamide gels (containing 7.5 M urea), after pre-running the gel for at least 30 minutes. The samples were electrophoresed for 1.5 h at 40 W limit. Afterwards, RNA was transferred to a presoaked Hybond-N+ membrane (303b, GE Healthcare) in 1× TBE buffer using electronic transfer system at 250 mA for 2 h in a cold room. RNA was then crosslinked to membrane via UV crosslinking (254 nm, 700 mJ), followed by pre-hybridization of the membrane at 60 °C in pre-hybridized buffer for 1 h. Then probes were added and hybridized overnight, after being labeled with [α-32P] CTP and GTP with Klenow DNA polymerase (New England Biolabs, Beverly, MA, USA) (Table S3). After washed three times with pre-warmed (60 °C) 2× SSC containing 0.5% SDS, the membrane was exposed to phosphor screen for one hour or overnight at room temperature and then scanned with phosphorimager (Typhoon).

## Electronic supplementary material

Supplemental information
