# Supplementary material for: SgRNA Expression of CRIPSR-Cas9 System Based on MiRNA Polycistrons as a Versatile Tool to Manipulate Multiple and Tissue-Specific Genome Editing
Source: Sci Rep. 2017 Jul 19;7:5795. doi: 10.1038/s41598-017-06216-w (PMC5517485; doi:10.1038/s41598-017-06216-w)
Supplement: Supplementary file 1 — Supplemental information [file 41598_2017_6216_MOESM1_ESM.pdf]

SgRNA Expression of CRIPSR-Cas9 System Based on MiRNA Polycistrons as a Versatile Tool to Manipulate Multiple and Tissue-Specific Genome Editing

Chen Xie[1,3,#], Yan-Lian Chen[2,#], Dong-Fang Wang[4], Yi-Lin Wang[5], Tian-Peng Zhang[2], Hui Li[3], Fu Liang[3], Yong Zhao[2,*], Guang-Ya Zhang[1,*]

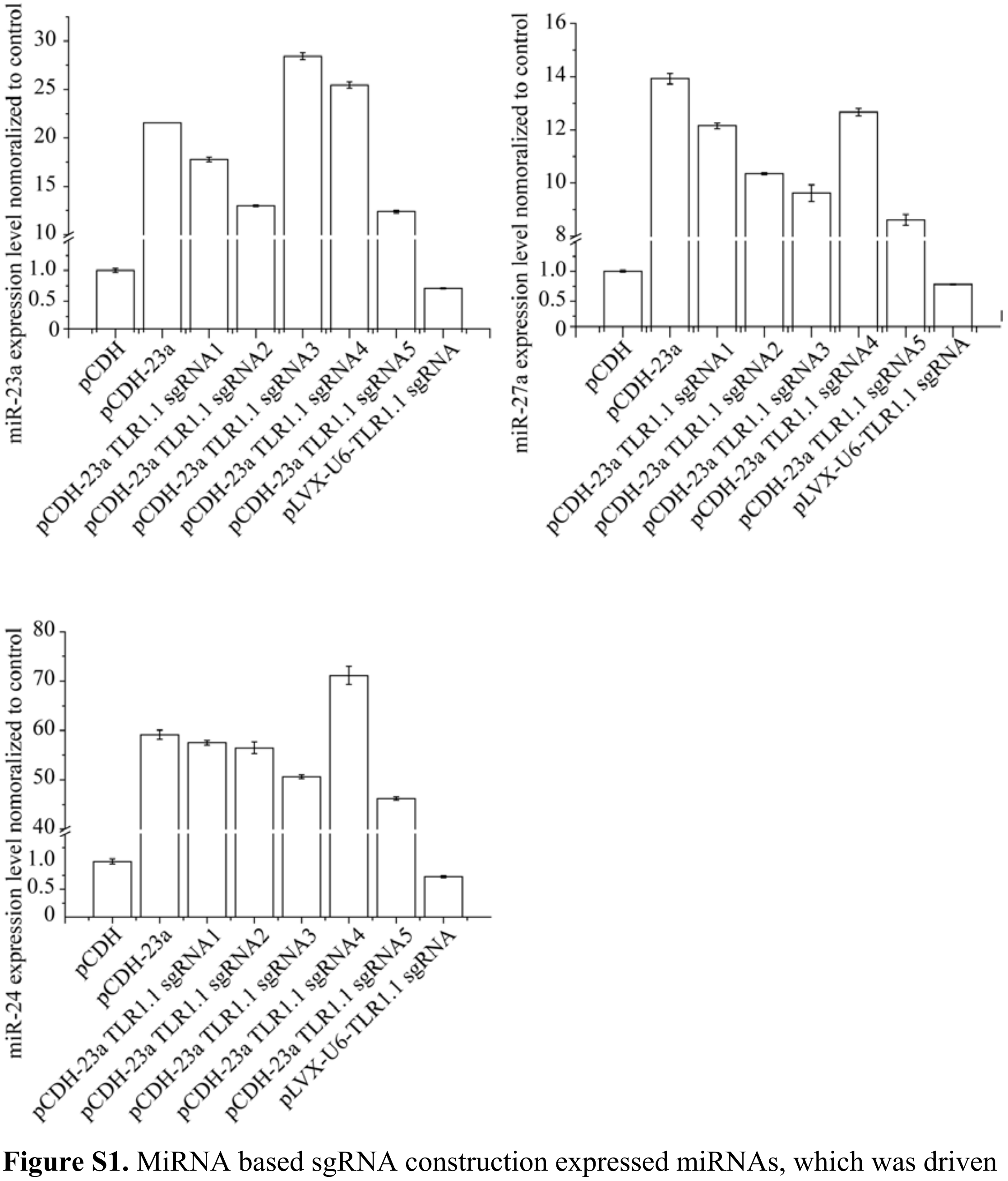

Figure S1. MiRNA based sgRNA construction expressed miRNAs, which was driven by RNA polymerase II promoter. (A-C) qRT-PCR was used to detect the expression level of mature miR-23a, miR-27a and miR-24.

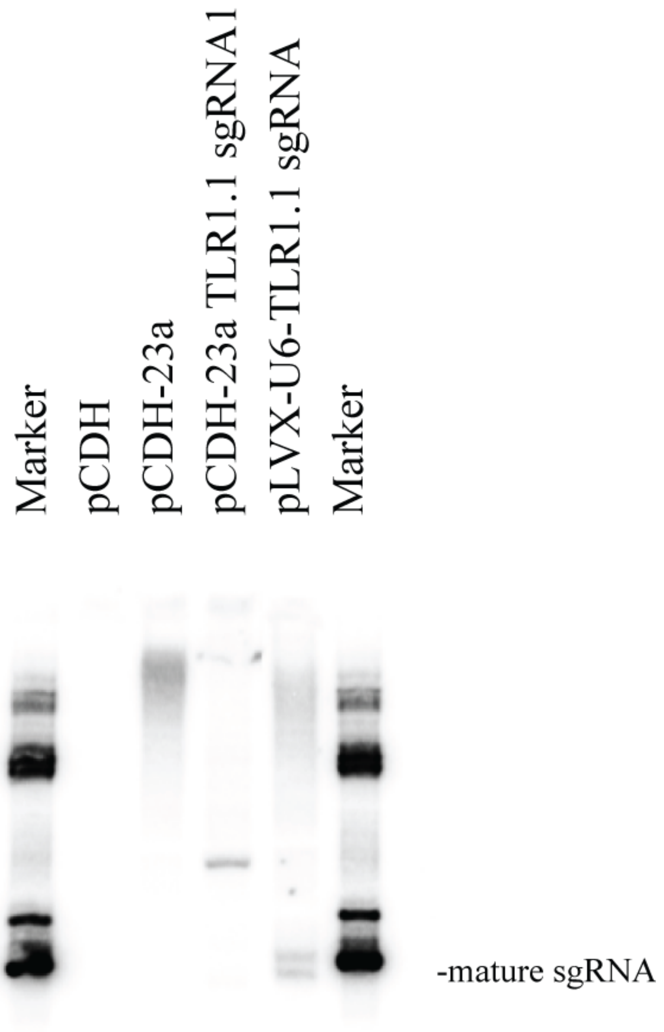

Figure S2. Northern blot was performed to analyze the processing of pCDH-23a TLR1.1 sgRNA1. Probes recognized the sequence of sgRNA.

Table S1. Primers used in plasmid construction and corresponding usage in cloning.

Name of plasmid	Primer name	Primer sequence	Methods
pCDH-EcoRI-XbaI sgRNA	EcoRI XbaI sgRNA 1 F	ctaggaattcaaaatCTAGAAATAGCAAGTTAAAAT	All of synthetic oligoes were mixed and phosphorylated by T4 PNK. Phosphorylated oligoes were annealed and ligated with pCDH-CMV-MCS-EF1-copGFP (cat# CD511B-1, SBI, System Biosciences, Mountain View, CA) digested by XbaI and BamHI.
	EcoRI XbaI sgRNA 1 R	GGACTAGCCTTATTTTAACTTGCTATTTCTAGATTTTGAATTC	
	BamHI 2 F	AAGGCTAGTCCGTTATCAACTTGAAAAAGTGGCACCGAGTCGGTGCTg	
	BamHI 2 R	GATCCAGCACCGACTCGGTGCCACTTTTTCAAGTTGATAAC	
pCDH-CMV-TLR1.1-sgRNA	CMV-TLR1.1 sgRNA F	AATTCTTATTTGCGTAGGGATAACAGTTTTAGAG	All of synthetic oligoes were annealed and ligated with pCDH-EcoRI-XbaI sgRNA digested by EcoRI and XbaI.
	CMV-TLR1.1 sgRNA R	CTAGCTCTAAAACTGTTATCCCTACGCAAATAAg	
pCDH-23a cluster	pCDH-miR-23a cluster F	acctccatagaagattctagaTGCTGTAGCCTCCTTGTCC	MiR-23a cluster was amplified from human genome DNA by using the primer illustrated above. Production of PCR was purified and digested by BamHI and XbaI then ligate with pCDH-CMV-MCS-EF1-copGFP also digested by XbaI and BamHI
	pCDH-miR-23a BamHI cluster R	gatccttgcggccgcggatccTCCAAGCATCAGCCCACCC	
pCDH-23a TLR1.1 sgRNA1	23a Before TLR1.1 XbaI FH	tttgacctccatagaagattctagaTTATTTGCGTAGGGATAACA	TLR1.1 sgRNA were amplified by primers described above, pCDH-CMV-TLR1.1-sgRNA serve as a template. Then the production of PCR were purified followed by ligation to XbaI-linearized plasmid pCDH-23a cluster based on homologous recombination using the NovoRec ® PCR Cloning Kit (Novoprotein Scientific Inc, Shanghai, China), yielding pCDH-23a TLR1.1 sgRNA1.
	23a Before TLR1.1 XbaI RH	catgcgggacaaggaggctacagcaGGAGCACCGACTCGGTGCCA	
pCDH-23a TLR1.1 sgRNA2	23a cluster D1 F	TGCCACCGAGGATGCTGTTATTTGCGTAGGGATAACAGTTTTAGAGCTAGAAATAGC	TLR1.1 sgRNA were amplified by primers described above, pCDH-CMV-TLR1.1-sgRNA serve as a template. Then the production of PCR were purified followed by ligation to XmaI-linearized plasmid pCDH-23a cluster based on homologous recombination using the NovoRec ® PCR Cloning Kit (Novoprotein Scientific Inc, Shanghai, China), yielding pCDH-23a TLR1.1 sgRNA1.
	23a cluster D1 R	TCTGCCACCCCGTCCCCGGGAGCACCGACTCGGTGCCACT	
pCDH-23a TLR1.1	23a cluster D3 F	TGCCACCGAGGATGCTGCCCGGGTTATTTGCGTAGGGATAACA	TLR1.1 sgRNA were amplified by primers described above, pCDH-CMV-TLR1.1-sgRNA serve as a template. Then the

sgRNA3	23a cluster D3 R	TCTCTGCCACCCCGTCAGCACCGACTCGGTGCCAC	production of PCR were purified followed by ligation to XmaI-linearized plasmid pCDH-23a cluster based on homologous recombination using the NovoRec ® PCR Cloning Kit (Novoprotein Scientific Inc, Shanghai, China), yielding pCDH-23a TLR1.1 sgRNA1.	
pCDH-23a TLR1.1 sgRNA4	pCDH-miR-23a cluster F	acctccatagaagattctagaTGCTGTAGCCTCCTTGTCC	P1 fragments were amplified with these two primers, pCDH-23a cluster serve as a template	Overlap extension (OE)-PCR was used to generate full length of miR-23a TLR1.1 sgRNA 4 with P1, P2 and P3 fragments. The full length fragments were digested with XbaI and BamHI and ligated into -CMV-MCS-EF1-copGFP that had been digested with the same enzymes to yield pCDH-23a TLR1.1 sgRNA4.
	23a cluster P1R	AGGATGGCAGGCAGACAGGC		
	23a cluster P2 TLR1.1 F	GCCTGTCTGCCTGCCATCCTTTATTTGCGTAGGGATAACA	P2 fragments were amplified with these two primers, pCDH-CMV-TLR1.1-sgRNA serve as a template	
	23a cluster P2 TLR1.1 R	GCCCAGGGAGGCCAGGCAGCGGAGCACCGACTCGGTGCCA		
	23a cluster P3 F	GCTGCCTGGCCTCCCTGGGC	P3 fragments were amplified with these two primers, pCDH-23a cluster serve as a template	
	pCDH-miR-23a BamHI cluster R	gatccttgcggccgcggatccTCCAAGCATCAGCCCACCC		
pCDH-23a TLR1.1 sgRNA5	24 after TLR1.1 BamHI FH	ttccctgggtgggctgatgcttggaTTATTTGCGTAGGGATAACA	TLR1.1 sgRNA were amplified by primers described above, pCDH-CMV-TLR1.1-sgRNA serve as a template. Then the production of PCR were purified followed by ligation to BamI-linearized plasmid pCDH-23a cluster based on homologous recombination using the NovoRec ® PCR Cloning Kit (Novoprotein Scientific Inc, Shanghai, China), yielding pCDH-23a TLR1.1 sgRNA1.	
	24 after TLR1.1 BamHI RH	cgcagatccttgcggccgcggatccAGCACCGACTCGGTGCCACT		
pLVX-U6-TLR1.1 sgRNA	BamHI TLR1.1 sgRNA F	cgcggatccTTATTTGCGTAGGGATAACA	TLR1.1 sgRNA were amplified by primers described above, pCDH-CMV-TLR1.1-sgRNA serve as a template. Then the production of PCR were digested with BamHI and EcoRI and ligated into pLVX-shRNA2 (Clontech, Palo Alto, CA, USA) that had been digested with the same enzymes to yield pLVX-U6-TLR1.1 sgRNA.	
	BamHI TLR1.1 sgRNA R	ggcGAATTCAAAAAAGCACCGACTCGGTGCCAC		
miR-23a sensor	23a-s1	TCGAATAAGGAAATCCCTCTAATGTGATCGATGGAAATCCCTCTAATGTGAT	All of synthetic oligoes were mixed and phosphorylated by T4 PNK. Phosphorylated oligoes were annealed and ligated with modified psi-check2 digested by XhoI and BamHI.	
	23a-s2	ACGCGTGGAAATCCCTCTAATGTGATTCACGGAAATCCCTCTAATGTGATC		

	23a-as1	ACGCGTATCACATTAGAGGGATTTCCATCGATCACATTAGAGGGATTTCCTTAT	
	23a-as2	GATCGATCACATTAGAGGGATTTCCGTGAATCACATTAGAGGGATTTCC	
miR-27a sensor	27a-s1	TCGAATAAGCGGAACTTACGACTGTGAACGATGCGGAACTTACGACTGTGAA	All of synthetic oligoes were mixed and phosphorylated by T4 PNK. Phosphorylated oligoes were annealed and ligated with modified psi-check2 digested by XhoI and BamHI.
	27a-s2	ACGCGTGCGGAACTTACGACTGTGAATCACGCGGAACTTACGACTGTGAAC	
	27a-as1	ACGCGTTTCACAGTCGTAAGTTCCGCATCGTTCACAGTCGTAAGTTCCGCTTAT	
	27a-as2	GATCGTTCACAGTCGTAAGTTCCGCGTGATTCACAGTCGTAAGTTCCGC	
miR-24-2 sensor	24-s1	TCGAATAACTGTTCCTGCTCCCTGAGCCACGATCTGTTCCTGCTCCCTGAGCCA	All of synthetic oligoes were mixed and phosphorylated by T4 PNK. Phosphorylated oligoes were annealed and ligated with modified psi-check2 digested by XhoI and BamHI.
	24-s2	ACGCGTCTGTTCCTGCTCCCTGAGCCATCACCTGTTCCTGCTCCCTGAGCCAC	
	24-as1	ACGCGTTGGCTCAGGGAGCAGGAACAGATCGTGGCTCAGGGAGCAGGAACAGTTAT	
	24-as2	GATCGTGGCTCAGGGAGCAGGAACAGGTGATGGCTCAGGGAGCAGGAACAG	
UTR-Lig4	UTR-hLIG4F	TCGATCTTCCTCAGCTAGAAAGAGAGAGAATGGCCTATGGAATTAAAGAAACTATGC	Designed shRNA target site in human Lig4 gene were synthetic as oligoes. A pair of oligoes were annealed and ligated with modified psi-check2 digested by XhoI and BamHI for detect the efficiency of miRNA based Lig4 shRNA.
	UTR-hLIG4R	gatcGCATAGTTTCTTTAATTCCATAGGCCATTCTCTCTCTTTCTAGCTGAGGAAGA	

Table S2. Primers used in qRT-PCR

Gene	Primer name	Primer sequences
miR-23a	miR-23a-3p RT	CTCAACTGGTGTCGTGGAGTCGGCAATTCAGTTGAGGGAAATCC
	miR-23a-3p F	CCAGCTGGGATCACATTGCCAGGGA
	miRNA universal Reverse primer	CTGGTGTCGTGGAGTCGGCAATT
miR-27a	miR-27a-3p RT	CTCAACTGGTGTCGTGGAGTCGGCAATTCAGTTGAGGCGGAACT
	miR-27a-3p F	CCAGCTGGGTTCACAGTGGCTAAGT
	miRNA universal Reverse primer	CTGGTGTCGTGGAGTCGGCAATT
miR-24-2	miR-24-3p RT	CTCAACTGGTGTCGTGGAGTCGGCAATTCAGTTGAGCTGTTCCT
	miR-24-3p F	CCAGCTGGGTGGCTCAGTTCAGCAG
	miRNA universal Reverse primer	CTGGTGTCGTGGAGTCGGCAATT
TLR1.1 sgRNA	TLR1.1 QRT F	GTTTTAGAGCTAGAAATAGCA
	PolyA Uni Reverse	GCGAGCACAGAATTAATACGACTCA
	PolyA RT	GCGAGCACAGAATTAATACGACTCACTATAGGTTTTTTTTTTTTTTTTTVN
Lig4	Lig4 QRT F	GCTTGCCCGAGGCCAGTTAA
	Lig4 QRT R	CTCCAGCATCTCCATGAGTTCCAGT
	DNA-PK QRT F	CCGGAAGTCACTCAACAGTA
	DNA-PK QRT Q	TCAACAGAGTAAGGTGCGAT
XRCC6	XRCC6 QRT F	GGAGCTGGATAATCCAGGTG
	XRCC6 QRT R	CACAGACCCACAGCACTTCA

Table S3. Oligonucleotides used to label probes

Primer name	Primer sequence
sgRNA probe template containing U	AGTTAAAAUAAGGCTAGUCCGTTAUCAACTUGAAAAAGUGGCACCGAGUCGGTGC
gRNA probe primer	GCACCGACTCGGTGC

Table S4. Oligonucleotides used in the restriction endonuclease digestion assay

Primer name	Primer sequence	Application
PGL3-Control F1	AATATTAAGGTACGGGAGGTACT	Use for I-SceI nuclease assay to evaluate the efficiency of miRNA based TLR1.1 sgRNA.
PGL3-Control R1	TATACATTAAGACGACTCGAAATCC	
HTP53 T7E1F3	ATGGAGGAGCCGCAGTCAGATCC	Use for PflMI nuclease assay to evaluate the efficiency of miRNA based p53 sgRNA.
HTP53 T7E1R6	AAATTTCCTTCCACTCGGATAAG	

Supplemental sequence

Sequence used in Figure 1

sgRNA sequence

>CMV Promoter
gtgatgcggttttggcagtacatcaatgggcgtggatagcggtttgactcacggggatttccaagtctccaccccattgacgtcaatgggagttt
gttttggcaccaaaatcaacgggactttccaaaatgtcgtaacaactccgccccattgacgcaaatgggcggtaggcgtgtacggtgggaggt
ctatataagcagagct
>U6 Promoter
gagggcctatttcccatgattccttcatatttgcatatacgatacaaggctgttagagagataattagaattaatttgactgtaaacacaaagatatt
agtacaaaatacgtgacgtagaaagtaataatttcttgggtagtttgcagttttaaaattatgttttaaaatggactatcatatgcttaccgtaacttga
aagtatttcgatttcttggctttatatatcttgtggaaaggac
>TLR1.1 sgRNA
ttatttgcgtagggataacagttttagagctagaaatagcaagttaaaataaggctagtccgttatcaacttgaaaaagtggcaccgagtcggtg
c
>pCDH-CMV TLR1.1 sgRNA
gtgatgcggttttggcagtacatcaatgggcgtggatagcggtttgactcacggggatttccaagtctccaccccattgacgtcaatgggagttt
gttttggcaccaaaatcaacgggactttccaaaatgtcgtaacaactccgccccattgacgcaaatgggcggtaggcgtgtacggtgggaggt
ctatataagcagagctcgtttagtgaaccgtcagatcgcctggagacgccatccacgctgttttgacctccatagaagattctaggaattcttattt
gcgtagggataacagttttagagctagaaatagcaagttaaaataaggctagtccgttatcaacttgaaaaagtggcaccgagtcggtgctgg
atcc
>pLVX-shRNA2 TLR1.1 sgRNA
gagggcctatttcccatgattccttcatatttgcatatacgatacaaggctgttagagagataattagaattaatttgactgtaaacacaaagatatt
agtacaaaatacgtgacgtagaaagtaataatttcttgggtagtttgcagttttaaaattatgttttaaaatggactatcatatgcttaccgtaacttga
aagtatttcgatttcttggctttatatatcttgtggaaaggacgaggatccttatttgcgtagggataacagttttagagctagaaatagcaagttaa
aataaggctagtccgttatcaacttgaaaaagtggcaccgagtcggtgcttttttgaattcta

Reporter sequence

>T2a
Gagggcagaggaagtcttctaacatgcggtgacgtggaggagaatccgggccct
>I-SceI nuclease site
attaccctgttatcccta
>TLR1.1 sgRNA target site
tgttatccctacgcaaataa
>DsRed2 sgRNA target site
caccgagttcatgcgcttca
>pGL3-control NHEJ V2
ATgacattaccctgttatccctacgcaaataaGGGACTATcaccgagttcatgcgcttcaagggagggcagaggaagtcttctaacat
gcggtgacgtggaggagaatccgggccct-firefly luciferase

Sequence used in Figure 3 & S1

sgRNA sequence

>CMV Promoter
Gtgatgcggttttggcagtacatcaatgggcgtggatagcggtttgactcacggggatttccaagtctccaccccattgacgtcaatgggagttt
gttttggcaccaaaatcaacgggactttccaaaatgtcgtaacaactccgccccattgacgcaaatgggcggtaggcgtgtacggtgggaggt
ctatataagcagagct
>TLR1.1 sgRNA
ttatttgcgtagggataacagttttagagctagaaatagcaagttaaaataaggctagtccgttatcaacttgaaaaagtggcaccgagtcggtg
c
>pre-miR-23a
ggccggctggggttcctggggatgggatttgcttcctgtcacaaatcacattgccagggatttccaaccgacc
>pre-miR-27a
ctgaggagcagggcttagctgcttgtgagcagggtccacaccaagtcgtgttcacagtggctaagttccgccccccag
>pre-miR-24-2
ctctgcctcccgtgcctactgagctgaaacacagttggtttgtgtacactggctcagttcagcaggaacaggg
> pCDH-23a
gtgatgcggttttggcagtacatcaatgggcgtggatagcggtttgactcacggggatttccaagtctccaccccattgacgtcaatgggagttt
gttttggcaccaaaatcaacgggactttccaaaatgtcgtaacaactccgccccattgacgcaaatgggcggtaggcgtgtacggtgggaggt
ctatataagcagagctcgtttagtgaaccgtcagatcgcctggagacgccatccacgctgttttgacctccatagaagattctagatcctgctgta
gcctccttgtcccgcatgggccctctaggtatctctgcctctccagtcctggggctggaacggagggcacagctaggctccagctccccgtgt
ggtggctcctgcatatgagaaaagagcttccctgtgatcaaaggaagcatctggggacctggaggggaggtgtccccaaatctcattacctcc
tttgctctctctctctttctcccctccaggtgccagcctctggccccgcccggtgccccccctcacccctgtgccacggccggctggggttcctgg
ggatgggatttgcttcctgtcacaaatcacattgccagggatttccaaccgaccctgagctctgccaccgaggatgctgcccggggacggggt
ggcagagaggccccgaagcctgtgcctggcctgaggagcagggcttagctgcttgtgagcagggtccacaccaagtcgtgttcacagtgg
ctaagttccgccccccaggccctcacctcctctggccttgccgcctgtcccctgctgccgcctgtctgcctgccatcctgctgcctggcctccct
gggctctgcctcccgtgcctactgagctgaaacacagttggtttgtgtacactggctcagttcagcaggaacagggtcaagccccccttggag
cctgcagcccctgccttccctgggtgggctgatgcttggaggatccgc
> pCDH-23a TLR1.1 sgRNA1
gtgatgcggttttggcagtacatcaatgggcgtggatagcggtttgactcacggggatttccaagtctccaccccattgacgtcaatgggagttt
gttttggcaccaaaatcaacgggactttccaaaatgtcgtaacaactccgccccattgacgcaaatgggcggtaggcgtgtacggtgggaggt
ctatataagcagagctcgtttagtgaaccgtcagatcgcctggagacgccatccacgctgttttgacctccatagaagattctagattatttgcgt
agggataacagttttagagctagaaatagcaagttaaaataaggctagtccgttatcaacttgaaaaagtggcaccgagtcggtgctcctgctg
tagcctccttgtcccgcatgggccctctaggtatctctgcctctccagtcctggggctggaacggagggcacagctaggctccagctccccgt
gtggtggctcctgcatatgagaaaagagcttccctgtgatcaaaggaagcatctggggacctggaggggaggtgtccccaaatctcattacct
cctttgctctctctctctttctcccctccaggtgccagcctctggccccgcccggtgccccccctcacccctgtgccacggccggctggggttcct
ggggatgggatttgcttcctgtcacaaatcacattgccagggatttccaaccgaccctgagctctgccaccgaggatgctgcccggggacgg
ggtggcagagaggccccgaagcctgtgcctggcctgaggagcagggcttagctgcttgtgagcagggtccacaccaagtcgtgttcacagt
ggctaagttccgccccccaggccctcacctcctctggccttgccgcctgtcccctgctgccgcctgtctgcctgccatcctgctgcctggcctc
cctgggctctgcctcccgtgcctactgagctgaaacacagttggtttgtgtacactggctcagttcagcaggaacagggtcaagccccccttg
gagcctgcagcccctgccttccctgggtgggctgatgcttggaggatccgc
> pCDH-23a TLR1.1 sgRNA2

gtgatgcggttttggcagtacatcaatgggcgtggatagcggtttgactcacggggatttccaagtctccaccccattgacgtcaatgggagttt
gttttggcaccaaaatcaacgggactttccaaaatgtcgtaacaactccgccccattgacgcaaatgggcggtaggcgtgtacggtgggaggt
ctatataagcagagctcgtttagtgaaccgtcagatcgcctggagacgccatccacgctgttttgacctccatagaagattctagatcctgctgta
gcctccttgtcccgcatgggccctctaggtatctctgcctctccagtcctggggctggaacggagggcacagctaggctccagctccccgtgt
ggtggctcctgcatatgagaaaagagcttccctgtgatcaaaggaagcatctggggacctggaggggaggtgtccccaaatctcattacctcc
tttgctctctctctctttctcccctccaggtgccagcctctggccccgcccggtgccccctcacccctgtgccacggccggctggggttcctgg
ggatgggatttgcttcctgtcacaaatcacattgccagggatttccaaccgaccctgagctctgccaccgaggatgctgttatttgcgtagggat
aacagttttagagctagaaatagcaagttaaaataaggctagtccgttatcaacttgaaaaagtggcaccgagtcggtgccccggggacggg
gtggcagagaggccccgaagcctgtgcctggcctgaggagcagggcttagctgcttgtgagcagggtccacaccaagtcgtgttcacagtg
gctaagttccgcccccccaggccctcacctcctctggccttgccgcctgtcccctgctgccgcctgtctgcctgccatcctgctgcctggcctcc
ctgggctctgcctcccgtgcctactgagctgaaacacagttggtttgtgtacactggctcagttcagcaggaacagggggtcaagccccccttgg
agcctgcagcccctgccttccctgggtgggctgatgcttggaggatccgc

> pCDH-23a TLR1.1 sgRNA3

gtgatgcggttttggcagtacatcaatgggcgtggatagcggtttgactcacggggatttccaagtctccaccccattgacgtcaatgggagttt
gttttggcaccaaaatcaacgggactttccaaaatgtcgtaacaactccgccccattgacgcaaatgggcggtaggcgtgtacggtgggaggt
ctatataagcagagctcgtttagtgaaccgtcagatcgcctggagacgccatccacgctgttttgacctccatagaagattctagatcctgctgta
gcctccttgtcccgcatgggccctctaggtatctctgcctctccagtcctggggctggaacggagggcacagctaggctccagctccccgtgt
ggtggctcctgcatatgagaaaagagcttccctgtgatcaaaggaagcatctggggacctggaggggaggtgtccccaaatctcattacctcc
tttgctctctctctctttctcccctccaggtgccagcctctggccccgcccggtgccccctcacccctgtgccacggccggctggggttcctgg
ggatgggatttgcttcctgtcacaaatcacattgccagggatttccaaccgaccctgagctctgccaccgaggatgctgcccgggttatttgcgt
agggataacagttttagagctagaaatagcaagttaaaataaggctagtccgttatcaacttgaaaaagtggcaccgagtcggtgcgacggg
gtggcagagaggccccgaagcctgtgcctggcctgaggagcagggcttagctgcttgtgagcagggtccacaccaagtcgtgttcacagtg
gctaagttccgcccccccaggccctcacctcctctggccttgccgcctgtcccctgctgccgcctgtctgcctgccatcctgctgcctggcctcc
ctgggctctgcctcccgtgcctactgagctgaaacacagttggtttgtgtacactggctcagttcagcaggaacagggggtcaagccccccttgg
agcctgcagcccctgccttccctgggtgggctgatgcttggaggatccgc

> pCDH-23a TLR1.1 sgRNA4

gtgatgcggttttggcagtacatcaatgggcgtggatagcggtttgactcacggggatttccaagtctccaccccattgacgtcaatgggagttt
gttttggcaccaaaatcaacgggactttccaaaatgtcgtaacaactccgccccattgacgcaaatgggcggtaggcgtgtacggtgggaggt
ctatataagcagagctcgtttagtgaaccgtcagatcgcctggagacgccatccacgctgttttgacctccatagaagattctagatcctgctgta
gcctccttgtcccgcatgggccctctaggtatctctgcctctccagtcctggggctggaacggagggcacagctaggctccagctccccgtgt
ggtggctcctgcatatgagaaaagagcttccctgtgatcaaaggaagcatctggggacctggaggggaggtgtccccaaatctcattacctcc
tttgctctctctctctttctcccctccaggtgccagcctctggccccgcccggtgccccctcacccctgtgccacggccggctggggttcctgg
ggatgggatttgcttcctgtcacaaatcacattgccagggatttccaaccgaccctgagctctgccaccgaggatgctgcccggggacggggt
ggcagagaggccccgaagcctgtgcctggcctgaggagcagggcttagctgcttgtgagcagggtccacaccaagtcgtgttcacagtgg
ctaagttccgcccccccaggccctcacctcctctggccttgccgcctgtcccctgctgccgcctgtctgcctgccatccttatttgcgtagggata
acagttttagagctagaaatagcaagttaaaataaggctagtccgttatcaacttgaaaaagtggcaccgagtcggtgcgctgcctggcctccc
tgggctctgcctcccgtgcctactgagctgaaacacagttggtttgtgtacactggctcagttcagcaggaacagggggtcaagccccccttgga
gcctgcagcccctgccttccctgggtgggctgatgcttggaggatccgc

> pCDH-23a TLR1.1 sgRNA5

gtgatgcggttttggcagtacatcaatgggcgtggatagcggtttgactcacggggatttccaagtctccaccccattgacgtcaatgggagttt
gttttggcaccaaaatcaacgggactttccaaaatgtcgtaacaactccgccccattgacgcaaatgggcggtaggcgtgtacggtgggaggt
ctatataagcagagctcgtttagtgaaccgtcagatcgcctggagacgccatccacgctgttttgacctccatagaagattctagatcctgctgta
gcctccttgtcccgcatgggccctctaggtatctctgcctctccagtcctggggctggaacggagggcacagctaggctccagctccccgtgt
ggtggctcctgcatatgagaaaagagcttccctgtgatcaaaggaagcatctggggacctggaggggaggtgtccccaaatctcattacctcc

tttgctctctctctctttctcccctccaggtgccagcctctggccccgcccggtgcccccctcacccctgtgccacggccggctggggttcctgg
ggatgggatttgcttcctgtcacaaatcacattgccagggatttccaaccgaccctgagctctgccaccgaggatgctgcccggggacggggt
ggcagagaggccccgaagcctgtgcctggcctgaggagcagggcttagctgcttgtgagcagggtccacaccaagtcgtgttcacagtgg
ctaagttccgcccccccaggccctcacctcctctggccttgccgcctgtcccctgctgccgcctgtctgcctgccatcctgctgcctggcctccct
gggctctgcctcccgtgcctactgagctgaaacacagttggtttgtgtacactggctcagttcagcaggaacagggggtcaagccccccttggag
cctgcagcccctgccttccctgggtgggctgatgcttggagttatttgcgtagggataacagttttagagctagaaatagcaagttaaaataagg
ctagtccgttatcaacttgaaaaagtggcaccgagtcggtgcgatccgc

>pLVX-shRNA2 TLR1.1 sgRNA

gagggcctatttcccatgattccttcatatttgcatatacgatacaaggctgttagagagataattagaattaatttgactgtaaacacaaagatatt
agtacaaaatacgtgacgtagaaagtaataatttcttgggtagtttgcagttttaaaattatgttttaaaatggactatcatatgcttaccgtaacttga
aagtatttcgatttcttggctttatatatcttgtggaaaggacgaggatccttatttgcgtagggataacagttttagagctagaaatagcaagttaa
aataaggctagtccgttatcaacttgaaaaagtggcaccgagtcggtgctttttgaattcta

Reporter sequence

>pGL3-control NHEJ V2

ATgacattaccctgttatccctacgcaaataaGGGACTATcaccgagttcatgcgcttcaagggagggcagaggaagtcttctaacat
gcggtgacgtggaggagaatccgggccct-firefly luciferase

Sequence used in Figure 4

sgRNA sequence

>hSynapsin Promoter
ctgcagagggccctgcgtatgagtgcaagtgggttttaggaccaggatgaggcggggtgggggtgcctacctgacgaccgaccccgaccc
actggacaagcacccaacccccattccccaaattgcgcatcccctatcagagagggggaggggaaacaggatgcggcgaggcgcgtgcg
cactgccagcttcagcaccgcggacagtgccttcgcccccgcctggcggcgcgcgccaccgccgcctcagcactga
>hGHintron1(AF264722.1)
gttcgaacaggtaagcgcccctaaaatccctttgggcacaatgtgtcctgaggggagaggcagcgacctgtagatgggacgggggcactaa
ccctcaggtttggggcttctgaatgtgagtatcgccatgtaagcccagtatttggccaatctcagaaagctcctggtccctggagggatggaga
gagaaaaacaaacagctcctggagcagggagagtgctggcctcttgctctccggctccctctgttgccctctggtttctccccaggtt
>EGFP
atggtgagcaagggcgaggagctgttcaccggggtggtgcccatcctggtcgagctggacggcgacgtaaacggccacaagttcagcgtg
tccggcgagggcgagggcgatgccacctacggcaagctgaccctgaagttcatctgcaccaccggcaagctgcccgtgccctggcccacc
ctcgtgaccaccctgacctacggcgtgcagtgcttcagccgctaccccgaccacatgaagcagcacgacttcttcaagtccgccatgcccga
aggctacgtccaggagcgcaccatcttcttcaaggacgacggcaactacaagacccgcgccgaggtgaagttcgagggcgacaccctggt
gaaccgcatcgagctgaagggcatcgacttcaaggaggacggcaacatcctggggcacaagctggagtacaactacaacagccacaacgt
ctatatcatggccgacaagcagaagaacggcatcaaggtgaacttcaagatccgccacaacatcgaggacggcagcgtgcagctcgccga
ccactaccagcagaacacccccatcggcgacggccccgtgctgctgcccgacaaccactacctgagcacccagtccgccctgagcaaaga
ccccaacgagaagcgcgatcacatggtcctgctggagttcgtgaccgccgccgggatcactctcggcatggacgagctgtacaag
>miR-24-2
ctctgcctcccgtgcctactgagctgaaacacagttggtttgtgtacactggctcagttcagcaggaacaggg
>DsRed2 sgRNA
caccgagttcatgcgcttca~~GTTT~~TAGAGCTAGAAATAGCAAGTTAAAATAAGGCTAGTCCGTTATCAACTTGAAAAAG
TGGCACCGAGTCGGTGCT
>WPRE
aatcaacctctggattacaaaatttgtgaaagattgactggtattcttaactatgttgctccttttacgctatgtggatacgctgctttaatgcctttgta
tcatgctattgcttcccgtatggctttcattttctcctccttgtataaatcctggttgctgtctctttatgaggagttgtggcccgttgtcaggcaacgtg
gcgtggtgtgcactgtgtttgctgacgcaacccccactggttggggcattgccaccacctgtcagctcctttccgggactttcgctttccccctcc
ctattgccacggcggaactcatcgccgcctgccttgcccgctgctggacaggggctcggctgttgggcactgacaattccgtggtgttgtcgg
ggaaatcatcgtcctttccttggctgctcgcctgtgttgccacctggattctgcgcgggacgtccttctgctacgtcccttcggccctcaatccag
cggaccttccttcccgcggcctgctgccggctctgcggcctcttccgcgtcttcgccttcgccctcagacgagtcggatctccctttgggccgc
ctccccgc
>SV40 poly(A) signal
taagatacattgatgagtttggacaaaccacaactagaatgcagtgaaaaaaatgctttatttgtgaaatttgtgatgctattgctttatttgtaacca
ttataagctgcaataaacaagtt
>hSynapsin DsRed sgRNA
ctgcagagggccctgcgtatgagtgcaagtgggttttaggaccaggatgaggcggggtgggggtgcctacctgacgaccgaccccgaccc
actggacaagcacccaacccccattccccaaattgcgcatcccctatcagagagggggaggggaaacaggatgcggcgaggcgcgtgcg
cactgccagcttcagcaccgcggacagtgccttcgcccccgcctggcggcgcgcgccaccgccgcctcagcactgaaggcgcgctgacg
tcactcgccggtcccccgcaaactccccttcccggccaccttggtcgcgtccgcgccgccgccggcccagccggaccgcaccacgcgag
gcgcgagatagggggcacgggcgcgaccatctgcgctgcggcgccggcgactcagcgctgcctcagtctgcggtgggcagcggagg

agtcgtgtcgtgcctgagagcgcagtcgaattcgcgtggtacctctagagtcgacgggaccgatccagcctccgtaccggttcgaacaggta
agcgcccctaaaatccctttgggcacaatgtgtcctgaggggagaggcagcgacctgtagatgggacgggggcactaaccctcaggtttgg
ggcttctgaatgtgagtatcgccatgtaagcccagtatttggccaatctcagaaagctcctggtccctggagggatggagagagaaaaacaaa
cagctcctggagcagggagagtgctggcctcttgctctccggctccctctgttgccctctggtttctccccaggttcgaatctagaggatccccg
ggtaccggtcgccaccatggtgagcaagggcgaggagctgttcaccggggtggtgcccatcctggtcgagctggacggcgacgtaaacg
gccacaagttcagcgtgtccggcgagggcgagggcgatgccacctacggcaagctgaccctgaagttcatctgcaccaccggcaagctgc
ccgtgccctggcccaccctcgtgaccaccctgacctacggcgtgcagtgcttcagccgctaccccgaccacatgaagcagcacgacttcttc
aagtccgccatgcccgaaggctacgtccaggagcgcaccatcttcttcaaggacgacggcaactacaagacccgcgccgaggtgaagttc
gagggcgacaccctggtgaaccgcatcgagctgaagggcatcgacttcaaggaggacggcaacatcctggggcacaagctggagtacaa
ctacaacagccacaacgtctatatcatggccgacaagcagaagaacggcatcaaggtgaacttcaagatccgccacaacatcgaggacggc
agcgtgcagctcgccgaccactaccagcagaacacccccatcggcgacggccccgtgctgctgcccgacaaccactacctgagcaccca
gtccgccctgagcaaagaccccaacgagaagcgcgatcacatggtcctgctggagttcgtgaccgccgccgggatcactctcggcatgga
cgagctgtacaagtccggagagcaaaagctgatttctgaggaggatctgggaggacccaagaagaagaggaaggtgtccaatttactgacc
gtacaccaaaatttgcctgcattaccggtcgatgcaacgagtgatgaggttcgcaagaacctgatggacatgttcagggatcgccaggcgtttt
ctgagcatacctggaaaatgcttctgtccgtttgccggtcgtgggcggcatggtgcaagttgaataaccggaaatggtttcccgcagaacctga
agatgttcgcgattatcttctatatcttcaggcgcgcggtctggcagtaaaaactatccagcaacatttgggccagctaaacatgcttcatcgtcg
gtccgggctgccacgaccaagtgacagcaatgctgtttcactggttatgcggcggatccgaaaagaaaacgttgatgccggtgaacgtgcaa
aacaggctctagcgttcgaacgcactgatttcgaccaggttcgttcactcatggaaaatagcgatcgctgccaggatatacgtaatctggcattt
ctggggattgcttataacaccctgttacgtatagccgaaattgccaggatcagggttaaagatatctcacgtactgacggtgggagaatgttaat
ccatattggcagaacgaaaacgctggttagcaccgcaggtgtagagaaggcacttagcctgggggtaactaaactggtcgagcgatggattt
ccgtctctggtgtagctgatgatccgaataactacctgttttgccgggtcagaaaaaatggtgttgccgcgccatctgccaccagccagctatca
actcgcgccctggaagggatttttgaagcaactcatcgattgatttacggcgctaaggatgactctggtcagagatacctggcctggtctggac
acagtgcccgtgtcggagccgcgcgagatatggcccgcgctggagtttcaataccggagatcatgcaagctggtggctggaccaatgtaaat
attgtcatgaactatatccgtaacctggatagtgaaacaggggcaatggtgcgcctgctggaagatggcgattagctcgatcaagcttccctca
cctcctctggccttgccgcctgtccc**ccctcacctcctctggccttgccgcctgtcccctgctgccgcctgtctgcctgccatcctgctgcctggc**
ctccctgggctctgcctcccgtgcctactgagctgaaacacagttggtttgtgtacactggctcagttcagcaggaacagggggtcaagcccc
cttggagcctgcagcccctgccttccctgggtgggctgatgcttggacaccgagttcatgcgcttca**gttttagagctagaaatagcaagtta**
aaataaggctagtccgttatcaacttgaaaaagtggcaccgagtcggtgctatcgataatcaacctctggattacaaaatttgtgaaagattga
ctggtattcttaactatgttgctccttttacgctatgtggatacgctgctttaatgcctttgtatcatgctattgcttcccgtatggctttcattttctcctcc
ttgtataaatcctggttgctgtctctttatgaggagttgtggcccgttgtcaggcaacgtggcgtggtgtgcactgtgtttgctgacgcaacccccca
ctggttggggcattgccaccacctgtcagctcctttccgggactttcgctttccccctccctattgccacggcggaactcatcgccgcctgccttg
cccgctgctggacaggggctcggctgttgggcactgacaattccgtggtgttgtcggggaaatcatcgtcctttccttggctgctcgcctgtgtt
gccacctggattctgcgcgggacgtccttctgctacgtcccttcggccctcaatccagcggaccttccttcccgcggcctgctgccggctctgc
ggcctcttccgcgtcttcgccttcgccctcagacgagtcggatctccctttgggccgcctccccgcatcgataccgtcgatcgaggccgcttcg
agcagacatgataagatacattgatgagtttggacaaaccacaactagaatgcagtgaaaaaaatgctttatttgtgaaatttgtgatgctattgct
ttatttgtaaccattataagctgcaataaacaagtt

>pLVX-shRNA2 TLR1.1 sgRNA

gagggcctatttcccatgattccttcatatttgcatatacgatacaaggctgttagagagataattagaattaatttgactgtaaacacaaagatatt
agtacaaaatacgtgacgtagaaagtaataatttcttgggtagtttgcagttttaaaattatgttttaaaatggactatcatatgcttaccgtaacttga
aagtatttcgatttcttggctttatatatcttgtggaaaggacgaggatccttatttgcgtagggataacagttttagagctagaaatagcaagttaa
aataaggctagtccgttatcaacttgaaaaagtggcaccgagtcggtgcttttttgaattcta

Reporter sequence

>T2a
Gagggcagaggaagtcttctaacatgcggtgacgtggaggagaatccgggccct
>I-SceI nuclease site
attaccctgttatcccta
>TLR1.1 sgRNA target site
tgttatccctacgcaaataa
>DsRed2 sgRNA target site
caccgagttcatgcgcttca
>pGL3-control NHEJ V2

ATgacattaccctgttatccctacgcaaataaGGGACTATcaccgagttcatgcgcttcaagggagggcagaggaagtcttctaacat
gcggtgacgtggaggagaatccgggccct-firefly luciferase

Sequence used in Figure 5

sgRNA sequence

>CMV Promoter
gacattgattattgactagttattaatagtaatcaattacggggtcattagttcatagcccatatatggagttccgcgttacataacttacggtaaatg
gcccgcctggctgaccgcccaacgacccccgcccattgacgtcaataatgacgtatgttcccatagtaacgccaatagggactttccattgac
gtcaatgggtggagtatttacggtaaactgcccacttggcagtacatcaagtgtatcatatgccaagtacgccccctattgacgtcaatgacggt
aaatggcccgcctggcattatgcccagtacatgaccttatgggactttcctacttggcagtacatctacgtattagtcatcgctattaccatggtga
tgcggttttggcagtacatcaatgggcgtggatagcggtttgactcacggggatttccaagtctccaccccattgacgtcaatgggagtttgtttt
ggcaccaaaatcaacgggactttccaaaatgtcgtaacaactccgccccattgacgcaaatgggcggtaggcgtgtacggtgggaggtctat
ataagcagagct
>shLig4
AGAGAGAGAATGGCCTATGGAAtagtgaagccacagatgtaTTCCATAGGCCATTCTCTCTCT
>TLR1.1 sgRNA
ttatttgcgtagggataacaGTTTTAGAGCTAGAAATAGCAAGTTAAAATAAGGCTAGTCCGTTAT
CAACTTGAAAAAGTGGCACCGAGTCGGTGCT
>bGH poly(A) signal
ctgtgccttctagttgccagccatctgttgtttgcccctcccccgtgccttccttgaccctggaaggtgccactcccactgtcctttcctaataaaat
gaggaaattgcatcgcattgtctgagtaggtgtcattctattctggggggtggggtggggcaggacagcaagggggaggattgggaagaca
atagcaggcatgctggggatgcggtgggctctatgg
>pcDNA3.1(-)-shNHEJ-TLR1.1 sgRNA
gacattgattattgactagttattaatagtaatcaattacggggtcattagttcatagcccatatatggagttccgcgttacataacttacggtaaatg
gcccgcctggctgaccgcccaacgacccccgcccattgacgtcaataatgacgtatgttcccatagtaacgccaatagggactttccattgac
gtcaatgggtggagtatttacggtaaactgcccacttggcagtacatcaagtgtatcatatgccaagtacgccccctattgacgtcaatgacggt
aaatggcccgcctggcattatgcccagtacatgaccttatgggactttcctacttggcagtacatctacgtattagtcatcgctattaccatggtga
tgcggttttggcagtacatcaatgggcgtggatagcggtttgactcacggggatttccaagtctccaccccattgacgtcaatgggagtttgtttt
ggcaccaaaatcaacgggactttccaaaatgtcgtaacaactccgccccattgacgcaaatgggcggtaggcgtgtacggtgggaggtctat
ataagcagagctctctggctaactagagaacccactgcttactggcttatcgaaattaatacgactcactatagggagacccaagctggctagc
gtttaaacgggccctctagaagcctctggccccgcccggtgccccctcacccctgtgccacggccggct*AGAGAGAGAATGGC*
*CTATGGAAtagtgaagccacagatgtaTTCCATAGGCCATTCTCTCTCT*aaccgaccctgagctctgccaccgagga
tgctgttatttgcgtagggataacaGTTTTAGAGCTAGAAATAGCAAGTTAAAATAAGGCTAGTCCGT
TATCAACTTGAAAAAGTGGCACCGAGTCGGTGCTcccgggccaatggatccgagctcggtaccaagcttaa
gtttaaaccgctgatcagcctcgactgtgccttctagttgccagccatctgttgtttgcccctcccccgtgccttccttgaccctggaaggtgcca
ctcccactgtcctttcctaataaaatgaggaaattgcatcgcattgtctgagtaggtgtcattctattctggggggtggggtggggcaggacagc
aagggggaggattgggaagacaatagcaggcatgctggggatgcggtgggctctatgg

>pLVX-shRNA2 TLR1.1 sgRNA
gagggcctatttcccatgattccttcatatttgcatatacgatacaaggctgttagagagataattagaattaatttgactgtaaacacaaagatatt
agtacaaaatacgtgacgtagaaagtaataatttcttgggtagtttgcagttttaaaattatgttttaaaatggactatcatatgcttaccgtaacttga
aagtatttcgatttcttggctttatatatcttgtggaaaggacgaggatccttatttgcgtagggataacagttttagagctagaaatagcaagttaa
aataaggctagtccgttatcaacttgaaaaagtggcaccgagtcggtgctttttttgaattcta

Reporter sequence

>SV40 promoter

Ctgaggcggaaagaaccagctgtggaatgtgtgtcagttagggtgtggaaagtccccaggctccccagcaggcagaagtatgcaaagcat
gcatctcaattagtcagcaaccaggtgtggaaagtccccaggctccccagcaggcagaagtatgcaaagcatgcatctcaattagtcagca
accatagtcccgcccctaactccgcccatcccgcccctaactccgcccagttccgcccattctccgccccatggctgactaattttttttatttat
gcagaggccgaggccgcctcggcctctgagctattccagaagtagtgaggaggcttttttggaggcctaggcttttgcaaa

>chimeric intron

gtaagtatcaaggttacaagacaggtttaaggagaccaatagaaactgggcttgtcgagacagagaagactcttgcgtttctgataggcacc
tattggtcttactgacatccactttgcctttctctccacag

>Renilla trunctC(+1)

Atggcttccaaggtgtacgaccccgagcaacgcaaacgcatgatcactgggcctcagtggtgggctcgctgcaagcaaatgaacgtgctgg
actccttcatcaactactatgattccgagaagcacgccgagaacgccgtgatttttctgcatggtaacgctgcctccagctacctgtggaggca
cgtcgtgcctcacatcgagcccgtggctagatgcatcatccctgatctgatcggaatgggtaagtccggcaagagcgggaatggctcatatc
gcctcctggatcactacaagtacctcaccgcttggttcgagctgctgaaccttccaaagaaaatcatctttgtgggccacgactgggggctt
gtctggcctttcactactcctacgagcaccaagacaagatcaaggccatcgtccatgctgagagtgtcgtggacgtgatcgagtcctgggacg
agtggcctgacatcgaggaggatatcgccctgatcaagagcgaagagggcgagaaaatggtgcttgagaataacttcttcgtcgagaccat
gctcccaagcaagatcatgcggaaactggagcctgaggagttcgctgcctacctggagccattcaaggagaagggcgaggttagacggcct
a

>I-SceI nuclease site

attaccctgttatccctа

>TLR1.1 sgRNA target site

tgttatccctacgcaaataa

>T2a(+3)

gagggcagaggaagtcttctaacatgcggtgacgtggaggagaatccgggccct

>Firefly luciferase(+3)

gccgatgctaagaacattaagaagggccctgctcccttctaccctctggaggatggcaccgctggcgagcagctgcacaaggccatgaaga
ggtatgccctggtgcctggcaccattgccttcaccgatgcccacattgaggtggacatcacctatgccgagtacttcgagatgtctgtgcgcct
ggccgaggccatgaagaggtacggcctgaacaccaaccaccgcatcgtggtgtgctctgagaactctctgcagttcttcatgccagtgctgg
gcgccctgttcatcggagtggccgtggcccctgctaacgacatttacaacgagcgcgagctgctgaacagcatgggcatttctcagcctaccg
tggtgttcgtgtctaagaagggcctgcagaagatcctgaacgtgcagaagaagctgcctatcatccagaagatcatcatcatggactctaag
accgactaccagggcttccagagcatgtacacattcgtgacatctcatctgcctcctggcttcaacgagtacgacttcgtgccagagtctttcg
acagggacaaaaccattgccctgatcatgaacagctctgggtctaccggcctgcctaagggcgtggccctgcctcatcgcaccgcctgtgtg
cgcttctctcacgcccgcgaccctattttcggcaaccagatcatccccgacaccgctattctgagcgtggtgccattccaccacggcttcggca
tgttcaccaccctgggctacctgatttgcggctttcgggtggtgctgatgtaccgcttcgaggaggagctgttcctgcgcagcctgcaagacta
caaaattcagtctgccctgctggtgccaaccctgttcagcttcttcgctaagagcaccctgatcgacaagtacgacctgtctaacctgcacgag
attgcctctggcggcgccccactgtctaaggaggtgggcgaagccgtggccaagcgctttcatctgccaggcatccgccagggctacggcct
gaccgagacaaccagcgccattctgattaccccagagggcgacgacaagcctggcgccgtgggcaaggtggtgccattcttcgaggccaag
gtggtggacctggacaccggcaagaccctgggagtgaaccagcgcggcgagctgtgtgtgcgcggccctatgattatgtccggctacgtga
ataaccctgaggccacaaacgccctgatcgacaaggacggctggctgcactctggcgacattgcctactgggacgaggacgagcacttctt
catcgtggaccgcctgaagtctctgatcaagtacaagggctaccaggtggccccagccgagctggagtctatcctgctgcagcaccctaaca
ttttcgacgccggagtggccggcctgcccgacgacgatgccggcgagctgcctgccgccgtcgtcgtgctggaacacggcaagaccatgac
cgagaaggagatcgtggactatgtggccagccaggtgacaaccgccaagaagctgcgcggcggagtggtgttcgtggacgaggtgcccaa
gggcctgaccggcaagctggacgcccgcaagatccgcgagatcctgatcaaggctaagaaaggcggcaagatcgccgtgtaa

>SV40 poly(A) signal

taagatacattgatgagtttggacaaaccacaactagaatgcagtgaaaaaaatgctttatttgtgaaatttgtgatgctattgctttatttgtaacca
ttataagctgcaataaacaagtt

>DSB repair

ctgaggcggaaagaaccagctgtggaatgtgtgtcagttagggtgtggaaagtccccaggctccccagcaggcagaagtatgcaaagcat
gcatctcaattagtcagcaaccaggtgtggaaagtccccaggctccccagcaggcagaagtatgcaaagcatgcatctcaattagtcagca
accatagtcccgcccctaactccgcccatcccgcccctaactccgcccagttccgcccattctccgccccatggctgactaattttttttatttat
gcagaggccgaggccgcctcggcctctgagctattccagaagtagtgaggaggcttttttggaggcctaggcttttgcaaaaagcttgattctt
ctgacacaacagtctcgaacttaagctgcagaagttggtcgtgaggcactgggcaggtaagtatcaaggttacaagacaggtttaaggaga
ccaatagaaactgggcttgtcgagacagagaagactcttgcgtttctgataggcacctattggtcttactgacatccactttgcctttctctcca
caggtgtccactcccagttcaattacagctcttaaggctagagtacttaatacgactcactataggctagccaccatggcttccaaggtgtacg
accccgagcaacgcaaacgcatgatcactgggcctcagtggtgggctcgctgcaagcaaatgaacgtgctggactccttcatcaactactat
gattccgagaagcacgccgagaacgccgtgatttttctgcatggtaacgctgcctccagctacctgtggaggcacgtcgtgcctcacatcgag
cccgtggctagatgcatcatccctgatctgatcggaatgggtaagtccggcaagagcgggaatggctcatatcgcctcctggatcactacaa
gtacctcaccgcttggttcgagctgctgaaccttccaaagaaaatcatctttgtgggccacgactgggggcttgtctggcctttcactactcct
acgagcaccaagacaagatcaaggccatcgtccatgctgagagtgtcgtggacgtgatcgagtcctgggacgagtggcctgacatcgagga
ggatatcgccctgatcaagagcgaagagggcgagaaaatggtgcttgagaataacttcttcgtcgagaccatgctcccaagcaagatcatg
cggaaactggagcctgaggagttcgctgcctacctggagccattcaaggagaagggcgaggttagacggcctaccgacattaccctgttat
ccctacgcaaataaCGGGACTATctggatccggtGagggcagaggaagtcttctaacatgcggtgacgtggaggagaatccgggccct
gccgatgctaagaacattaagaagggccctgctcccttctaccctctggaggatggcaccgctggcgagcagctgcacaaggccatgaaga
ggtatgccctggtgcctggcaccattgccttcaccgatgcccacattgaggtggacatcacctatgccgagtacttcgagatgtctgtgcgcct
ggccgaggccatgaagaggtacggcctgaacaccaaccaccgcatcgtggtgtgctctgagaactctctgcagttcttcatgccagtgctgg
gcgccctgttcatcggagtggccgtggcccctgctaacgacatttacaacgagcgcgagctgctgaacagcatgggcatttctcagcctaccg
tggtgttcgtgtctaagaagggcctgcagaagatcctgaacgtgcagaagaagctgcctatcatccagaagatcatcatcatggactctaag
accgactaccagggcttccagagcatgtacacattcgtgacatctcatctgcctcctggcttcaacgagtacgacttcgtgccagagtctttcg
acagggacaaaaccattgccctgatcatgaacagctctgggtctaccggcctgcctaagggcgtggccctgcctcatcgcaccgcctgtgtg
cgcttctctcacgcccgcgaccctatttcggcaaccagatcatccccgacaccgctattctgagcgtggtgccattccaccacggcttcggca
tgttcaccaccctgggctacctgatttgcggctttcgggtggtgctgatgtaccgcttcgaggaggagctgttcctgcgcagcctgcaagacta
caaaattcagtctgccctgctggtgccaaccctgttcagcttcttcgctaagagcaccctgatcgacaagtacgacctgtctaacctgcacgag
attgcctctggcggcgccccactgtctaaggaggtgggcgaagccgtggccaagcgctttcatctgccaggcatccgccagggctacggcct
gaccgagacaaccagcgccattctgattaccccagagggcgacgacaagcctggcgccgtgggcaaggtggtgccattcttcgaggccaag
gtggtggacctggacaccggcaagaccctgggagtgaaccagcgcggcgagctgtgtgtgcgcggccctatgattatgtccggctacgtga
ataaccctgaggccacaaacgccctgatcgacaaggacggctggctgcactctggcgacattgcctactgggacgaggacgagcacttctt
catcgtggaccgcctgaagtctctgatcaagtacaagggctaccaggtggccccagccgagctggagtctatcctgctgcagcaccctaaca
ttttcgacgccggagtggccggcctgcccgacgacgatgccggcgagctgcctgccgccgtcgtcgtgctggaacacggcaagaccatgac
cgagaaggagatcgtggactatgtggccagccaggtgacaaccgccaagaagctgcgcggcggagtggtgttcgtggacgaggtgcccaa
gggcctgaccggcaagctggacgcccgcaagatccgcgagatcctgatcaaggctaagaaaggcggcaagatcgccgtgtaataattcta
gagtcggggcggccggccgcttcgagcagacatgataagatacattgatgagtttggacaaaccacaactagaatgcagtgaaaaaaatg
ctttatttgtgaaatttgtgatgctattgctttatttgtaaccattataagctgcaataaacaagtt

>HR F for Renilla luciferase donor
GCCTCACATCGAGCCCGTGGCTAGATGCATCATCCCTGATCTGATCGGAATGGGTAAGTCCGGCAAGAGCGG
GAATGGCTCATATCGCCTCCTGGATCACTACAAGTACCTCACCGCTTGGTTCGAGCTGCTGAACCTTCCAAAG
AAAATCATCTTTGTGGGCCACGACTGGGGGGCTTGTCTGGCCTTTCACTACTCCTACGAGCACCAAGACAA
GATCAAGGCCATCGTCCATGCTGAGAGTGTCGTGGACGTGATCGAGTCCTGGGACGAGTGGCCTGACATCG
AGGAGGATATCGCCCTGATCAAGAGCGAAGAGGGCGAGAAAATGGTGCTTGAGAATAACTTCTTCGTCGA
GACCATGCTCCCAAGCAAGATCATGCGGAAACTGGAGCCTGAGGAGTTCGCTGCCTACCTGGAGCCATTCA
AGGAGAAGGGCGAGGTTAGACGGCCTA

>hRluc trunctC
CCCTCTCCTGGCCTCGCGAGATCCCTCTCGTTAAGGGAGGCAAGCCCGACGTCGTCCAGATTGTCCGCAACT
ACAACGCCTACCTTCGGGCCAGCGACGATCTGCCTAAGATGTTCATCGAGTCCGACCCTGGGTTCTTTTCCA
ACGCTATTGTCGAGGGAGCTAAGAAGTTCCCTAACACCGAGTTCGTGAAGGTGAAGGGCCTCCACTTCAGC
CAGGAGGACGCTCCAGATGAAATGGGTAAGTACATCAAGAGCTTCGTGGAGCGCGTGCTGAAGAACGAGC
AG

>HR R for Firefly luciferase donor
GCCGATGCTAAGAACATTAAGAAGGGCCCTGCTCCCTTCTACCCTCTGGAGGATGGCACCGCTGGCGAGCA
GCTGCACAAGGCCATGAAGAGGTATGCCCTGGTGCCTGGCACCATTGCCTTCACCGATGCCCACATTGAGG
TGGACATCACCTATGCCGAGTACTTCGAGATGTCTGTGCGCCTGGCCGAGGCCATGAAGAGGTACGGCCTG
AACACCAACCACCGCATCGTGGTGTGCTCTGAGAACTCTCTGCAGTTCTTCATGCCAGTGCTGGGCGCCCTG
TTCATCGGAGTGGCCGTGGCCCCTGCTAACGACATTTACAACGAGCGCGAGCTGCTGAACAGCATGGGCAT
TTCTCAGCCTACCGTGGTGTTCGTGTCTAAGAAGGGCCTGCAGAAGATCCTGAACGTGCAGAAGAAGCTGC
CTATCATCCAGAAGATCATCATCATGGACTCTAAGACCGACTACCAGGGCTTCCAGAGCATGTACACATTCGT
GACATCTCATCTGCCTCCTGGCTTCAACGAGTACGACTTCGTGCCAGAGTCTTTCGACAGGGACAAAACCAT
TGCCCTGATCATGAACAGCTCTGGGTCTACCGGCCT

>Donor
GCCTCACATCGAGCCCGTGGCTAGATGCATCATCCCTGATCTGATCGGAATGGGTAAGTCCGGCAAGAGCGG
GAATGGCTCATATCGCCTCCTGGATCACTACAAGTACCTCACCGCTTGGTTCGAGCTGCTGAACCTTCCAAAG
AAAATCATCTTTGTGGGCCACGACTGGGGGGCTTGTCTGGCCTTTCACTACTCCTACGAGCACCAAGACAA
GATCAAGGCCATCGTCCATGCTGAGAGTGTCGTGGACGTGATCGAGTCCTGGGACGAGTGGCCTGACATCG
AGGAGGATATCGCCCTGATCAAGAGCGAAGAGGGCGAGAAAATGGTGCTTGAGAATAACTTCTTCGTCGA
GACCATGCTCCCAAGCAAGATCATGCGGAAACTGGAGCCTGAGGAGTTCGCTGCCTACCTGGAGCCATTCA
AGGAGAAGGGCGAGGTTAGACGGCCTACCCTCTCCTGGCCTCGCGAGATCCCTCTCGTTAAGGGAGGCAA
GCCCGACGTCGTCCAGATTGTCCGCAACTACAACGCCTACCTTCGGGCCAGCGACGATCTGCCTAAGATGTT
CATCGAGTCCGACCCTGGGTTCTTTTCCAACGCTATTGTCGAGGGAGCTAAGAAGTTCCCTAACACCGAGTT
CGTGAAGGTGAAGGGCCTCCACTTCAGCCAGGAGGACGCTCCAGATGAAATGGGTAAGTACATCAAGAGC
TTCGTGGAGCGCGTGCTGAAGAACGAGCAGTAAAtatccctacgcaaataACGGGACTATctggatccggtgagggca
gaggaagtcttctaacatgcggtgacgtggaggagaatccgggccctGCCGATGCTAAGAACATTAAGAAGGGCCCTGCTC
CCTTCTACCCTCTGGAGGATGGCACCGCTGGCGAGCAGCTGCACAAGGCCATGAAGAGGTATGCCCTGGTG
CCTGGCACCATTGCCTTCACCGATGCCCACATTGAGGTGGACATCACCTATGCCGAGTACTTCGAGATGTCTG
TGCGCCTGGCCGAGGCCATGAAGAGGTACGGCCTGAACACCAACCACCGCATCGTGGTGTGCTCTGAGAA
CTCTCTGCAGTTCTTCATGCCAGTGCTGGGCGCCCTGTTCATCGGAGTGGCCGTGGCCCCTGCTAACGACAT
TTACAACGAGCGCGAGCTGCTGAACAGCATGGGCATTTCTCAGCCTACCGTGGTGTTCGTGTCTAAGAAGG
GCCTGCAGAAGATCCTGAACGTGCAGAAGAAGCTGCCTATCATCCAGAAGATCATCATCATGGACTCTAAGA
CCGACTACCAGGGCTTCCAGAGCATGTACACATTCGTGACATCTCATCTGCCTCCTGGCTTCAACGAGTACGA

CTTCGTGCCAGAGTCTTTCGACAGGGACAAAACCATTGCCCTGATCATGAACAGCTCTGGGTCTACCGGCCT

Sequence used in Figure 6

sgRNA sequence

> CMV Promoter

gtgatgcggttttggcagtacatcaatgggcgtggatagcggtttgactcacggggatttccaagtctccaccccattgacgtcaatgggagttt
gttttggcaccaaaatcaacgggactttccaaaatgtcgtaacaactccgccccattgacgcaaatgggcggtaggcgtgtacggtgggaggt
ctatataagcagagct

>TLR1.1 sgRNA

ttatttgcgtagggataacagttttagagctagaaatagcaagttaaaataaggctagtccgttatcaacttgaaaaagtggcaccgagtcggtg
c

>p53 sgRNA

ccccttgccgtcccaagcaaGTTTTAGAGCTAGAAATAGCAAGTTAAAATAAGGCTAGTCCGTTATCAACTTGAAAAAG
TGGCACCGAGTCGGTGCT

>pre-miR-23a

ggccggctggggttcctggggatgggatttgcttcctgtcacaaatcacattgccagggatttccaaccgacc

>pre-miR-27a

ctgaggagcagggcttagctgcttgtgagcagggtccacaccaagtcgtgttcacagtggctaagttccgccccccag

>pre-miR-24-2

ctctgcctcccgtgcctactgagctgaaacacagttggtttgtgtacactggctcagttcagcaggaacaggg

> pCDH- miR-23a-TLR1.1 sgRNA-p53 sgRNA

gtgatgcggttttggcagtacatcaatgggcgtggatagcggtttgactcacggggatttccaagtctccaccccattgacgtcaatgggagttt
gttttggcaccaaaatcaacgggactttccaaaatgtcgtaacaactccgccccattgacgcaaatgggcggtaggcgtgtacggtgggaggt
ctatataagcagagctcgtttagtgaaccgtcagatcgcctggagacgccatccacgctgttttgacctccatagaagattctagatcctgctgta
gcctccttgtcccgcatgggccctctaggtatctctgcctctccagtcctggggctggaacggagggcacagctaggctccagctccccgtgt
ggtggctcctgcatatgagaaaagagcttccctgtgatcaaaggaagcatctggggacctggaggggaggtgtccccaaatctcattacctcc
tttgctctctctctctttctcccctccaggtgccagcctctggccccgcccggtgccccctcacccctgtgccacggccggctggggttcctgg
ggatgggatttgcttcctgtcacaaatcacattgccagggatttccaaccgaccctgagctctgccaccgaggatgctgttatttgcgtagggat
aacagttttagagctagaaatagcaagttaaaataaggctagtccgttatcaacttgaaaaagtggcaccgagtcggtgccccggggacggg
gtggcagagaggccccgaagcctgtgcctggcctgaggagcagggcttagctgcttgtgagcagggtccacaccaagtcgtgttcacagtg
gctaagttccgccccccaggccctcacctcctctggccttgccgcctgtcccctgctgccgcctgtctgcctgccatcctgctgcctggcctcc
ctgggctctgcctcccgtgcctactgagctgaaacacagttggtttgtgtacactggctcagttcagcaggaacagggtcaagccccccttgg
agcctgcagcccctgccttccctgggtgggctgatgcttggaccccttgccgtcccaagcaaGTTTTAGAGCTAGAAATAGCAAGTT
AAAATAAGGCTAGTCCGTTATCAACTTGAAAAAGTGGCACCGAGTCGGTGCT

Reporter sequence

>pGL3-control NHEJ V2

ATgacattaccctgttatccctacgcaaataaGGGACTATcaccgagttcatgcgcttcaagggagggcagaggaagtcttctaacat
gcggtgacgtggaggagaatccgggccct-firefly luciferase

>T2a

Gagggcagaggaagtcttctaacatgcggtgacgtggaggagaatccgggccct

>I-SceI nuclease site

attaccctgttatcccta

>TLR1.1 sgRNA target site

tgttatccctacgcaaataa

>DsRed2 sgRNA target site

caccgagttcatgcgcttca
